# Squaring the Hexagon: Multiply Twinned Plasmonic Magnesium Nanocubes

**DOI:** 10.1021/acs.nanolett.6c04201

**Published:** 2026-10-19

**Authors:** Christina Boukouvala, Andrey Ten, Vladimir Lomonosov, Emilie Ringe

**Affiliations:** † Department of Materials Science and Metallurgy, 2152 University of Cambridge, 27 Charles Babbage Road, Cambridge CB3 0FS, United Kingdom; ‡ National Center for Electron Microscopy, Molecular Foundry, Lawrence Berkeley National Laboratory, Berkeley, California 94720, United States; § Department of Earth Sciences, University of Cambridge, Downing Street, Cambridge CB2 3EQ, United Kingdom

**Keywords:** Alternative plasmonic materials, magnesium nanoparticles, multiply twinned nanoparticles, localized surface plasmon resonance, metallic nanocubes

## Abstract

Magnesium, a promising alternative for sustainable nanophotonics, crystallizes in a hexagonal close-packed (HCP) system, unlike the face-centered cubic (FCC) structures of gold, silver, copper, and aluminum. We report the high-yield colloidal synthesis of an unexpected shape in an HCP system: cubes. Nanocubes with narrow size distributions were produced from colloidal reduction of a magnesium precursor in the presence of a sodium salt nucleating agent. Electron microscopy and diffraction reveal multiple twinning leading to the formation of six single-crystal units within the nanocubes. A self-limiting, sub-10 nm passivating oxide layer protects the metallic core against further oxidation in air and organic solvents. Strong, multimode plasmonic responses were observed using bulk extinction spectroscopy, single-particle scattering correlated with electron microscopy, and electron energy loss spectroscopy; experimental results were corroborated by numerical simulations. These findings expand the crystallographic boundaries of non-FCC plasmonics and establish magnesium nanocubes as versatile building blocks for nanophotonic applications.

## Graphical abstract

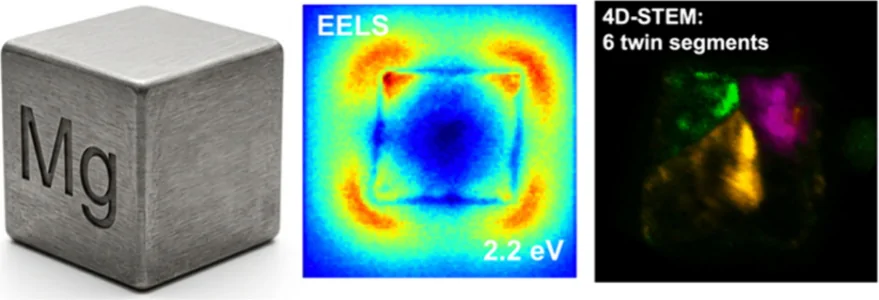

A defining feature of nanotechnology is the strong dependence of properties on the particle shape. In catalysis, for instance, different shapes imply different crystal facets that in turn dictate varying levels of reactivity.^1,2^ Similarly, in drug delivery, nanoparticle (NP) shape influences cellular uptake,^3^ while in sensing applications, sharp geometric features can amplify spectroscopic signals.^4,5^ This ability to tune properties drives intense research efforts toward precisely controlling the NP shape across a variety of materials.

Shape also influences nanoscale optical properties, particularly how the free electrons of a metallic NP interact with electromagnetic fields, leading to coherent, resonant oscillations known as localized surface plasmon resonances (LSPRs). Sharp structural features increase field localization, whereas the aspect ratio (shape) modifies the resonance energy. Further, the resonant mode geometry and degeneracy are functions of NP shape.

One of the simplest, high-symmetry shapes commonly obtained for plasmonic metals is a cube. Previous reports include nanocubes of silver (Ag),^6^ gold (Au),^7^ copper (Cu),^8^ and, more recently, aluminum (Al);^9^ all of them display strong LSPRs at energies characteristic of the constituent metals. Their formation is simple to explain: these metals crystallize in a face-centered-cubic (FCC) lattice, for which a single crystal predominantly displaying {100} facets creates a cubic shape.

Magnesium (Mg) is a new addition to the plasmonics toolbox.^10,11^ Although its plasmon quality factor is not as high as Ag’s, Mg compensates by sustaining LSPRs across the entire UV–vis–NIR spectrum due to the absence of interband transitions in these regions. Additionally, Mg is highly biocompatible, making it a promising candidate for biomedical applications such as photothermal therapy.^12,13^ Lastly, Mg exhibits excellent air stability due to a naturally forming, self-limiting oxide layer.^14^

Unlike Ag, Au, Cu, and Al, all FCC metals, Mg has a hexagonal close-packed (HCP) lattice. This lattice, space group *P*6_3_/*mmc*, and its intrinsic ability to twin along a plethora of twin planes (as compared to just one for FCC) bestow inherent complexity to Mg crystal growth. This complexity, while making syntheses challenging, provides opportunities to create unique plasmonic shapes such as singly twinned folded hexagons forming kite-, tent-, chair-, and taco-like structures.^15^

In this work, we report that multiply twinned Mg forms nanocubes reproducibly and in a high yield. We examined their composition, oxide shell, crystallography, and plasmonic properties. We show via both optical and electron spectroscopies that Mg nanocubes can support multiple LSPRs in the visible range. Experimental results are corroborated by numerical simulations, which also allow for resonance mode assignment and the effect of the oxide shell and the substrate to be explored numerically.

Nucleating agents can promote heterogeneous nucleation and influence the subsequent growth and shape of colloidal crystals. In this study, alkali metal salts were selected as nucleation agents in the synthesis of Mg NPs based on (1) their almost zero solubility in the reaction medium (THF) and (2) their cation having sufficiently large negative reduction potential (E^0^ of −2.71, −2.93, and −3.04 V for Na^+^, K^+^, and Li^+^, respectively) such that it is unlikely to react with the Mg^2+^ precursor or, via galvanic replacement, with the Mg NP. Briefly, Mg NPs are produced from the reduction of 1.75 mmol (0.14 M) of a Mg precursor (di-n-butyl Mg, MgBu_2_) by a premixed Li (4.0 mmol, 0.32 M) and naphthalene (2.0 mmol, 0.16 M) THF solution forming the purple dianion Li_2_Napht.^16^

As is, the reduction of MgBu_2_ by Li_2_Napht produced plasmonic, spheroidal Mg NPs (Figure S1) consistent with previous reports.^16,17^ However, in the presence of additive-specific amounts of insoluble sodium salts nearing 1:10 salt:Mg^2+^ (0.24 mmol bicarbonate, 0.21 mmol sulfate or 0.10 mmol dodecyl sulfate), Mg nanocubes consisting of a metallic Mg core and a thin MgO layer were formed (Figure 1, S2). The cubic shape of the samples produced was confirmed by scanning electron microscopy (SEM, Figure 1, Figure S2), high resolution transmission EM (HRTEM, Figure S3), and high angle annular dark field STEM tilt series (HAADF-STEM, Figures S4–S5). In addition to nanocubes, a small fraction of very thin hexagonal platelets was produced; these were in the smallest proportion for reactions with sodium dodecyl sulfate (SDS). The addition of lithium carbonate did not noticeably alter the reaction outcome (spheroidal particles). Meanwhile, the reduction of MgBu_2_ in the presence of potassium bicarbonate (0.20 mmol) produced coral-like Mg NPs with strong infrared absorption (Figure S6) currently under further investigation; these results will be reported elsewhere. Here, we focus on Mg nanocubes, a crystallographically puzzling shape arising from the HCP lattice of Mg.

**Figure 1:**
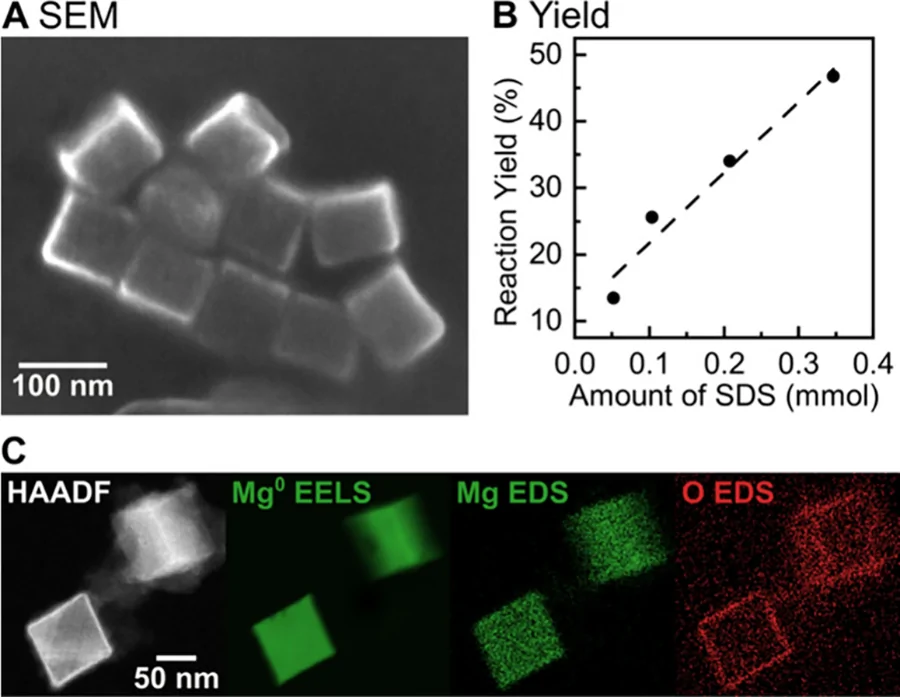
The presence of insoluble Na-containing salts as nucleating agents leads to the formation of metallic Mg nanocubes surrounded by a native oxide layer. (A) Representative SEM image (additional images in Figure S2), (B) solid Mg yield as a function of the amount of sodium dodecyl sulfate (SDS) added, and (C) HAADF-STEM image, STEM-EELS map of the bulk plasmon resonance of Mg showing metallic response, and STEM-EDS maps of Mg and O K_α_.

The amount of Na-containing salt added, well beyond its (very low) solubility^18^ limit, significantly manipulates the reaction outcome and kinetics, as suggested by an early result from Rieke and Bales.^19^ Spheroidal NPs, nanocubes, and a bimodal sample consisting of thin hexagonal platelets and small spheroidal NPs were produced from 0.14 M Mg precursor (1.75 mmol) in the presence of 0.052, 0.10, and 0.21–0.46 mmol of SDS, respectively (Figures S2, S7). In all cases, powder of nucleating agent was recovered along with dark Mg NPs (Figure S8), confirming their low solubility under the reaction conditions. While this contamination precluded bulk analysis by powder X-ray diffraction, we later show by single-particle electron diffraction that the nanocubes and hexagonal structures all crystallize as HCP Mg. The yield of Mg NPs (measured as the Mg in centrifuged solids over the total Mg in the precursor) after 10 min of reaction increased sharply with the amount of SDS added (Figure 1B). This dramatic change in the reaction rate occurs despite the concentration of dissolved SDS being equal (saturated) in all reactions, indicating its nucleating-agent behavior.

To investigate MgBu_2_ reduction in the presence of SDS, aliquots were taken from the reaction at different times under an inert atmosphere (Ar) and quenched immediately in an excess of acetone. Sub-40 nm cube-like Mg NPs were formed after 3 min of reaction (Figure S9); size and shape characterization was precluded by excessive contamination typical for the initial reaction stages in Mg NPs colloids.^16,20^ Aliquots taken after 6 and 10 min revealed an abundance of Mg nanocubes with average sizes and standard deviations of 48 ± 4 and 84 ± 5 nm, respectively (N = 28 and 55, Figure 2A,B, Figure S10).

**Figure 2:**
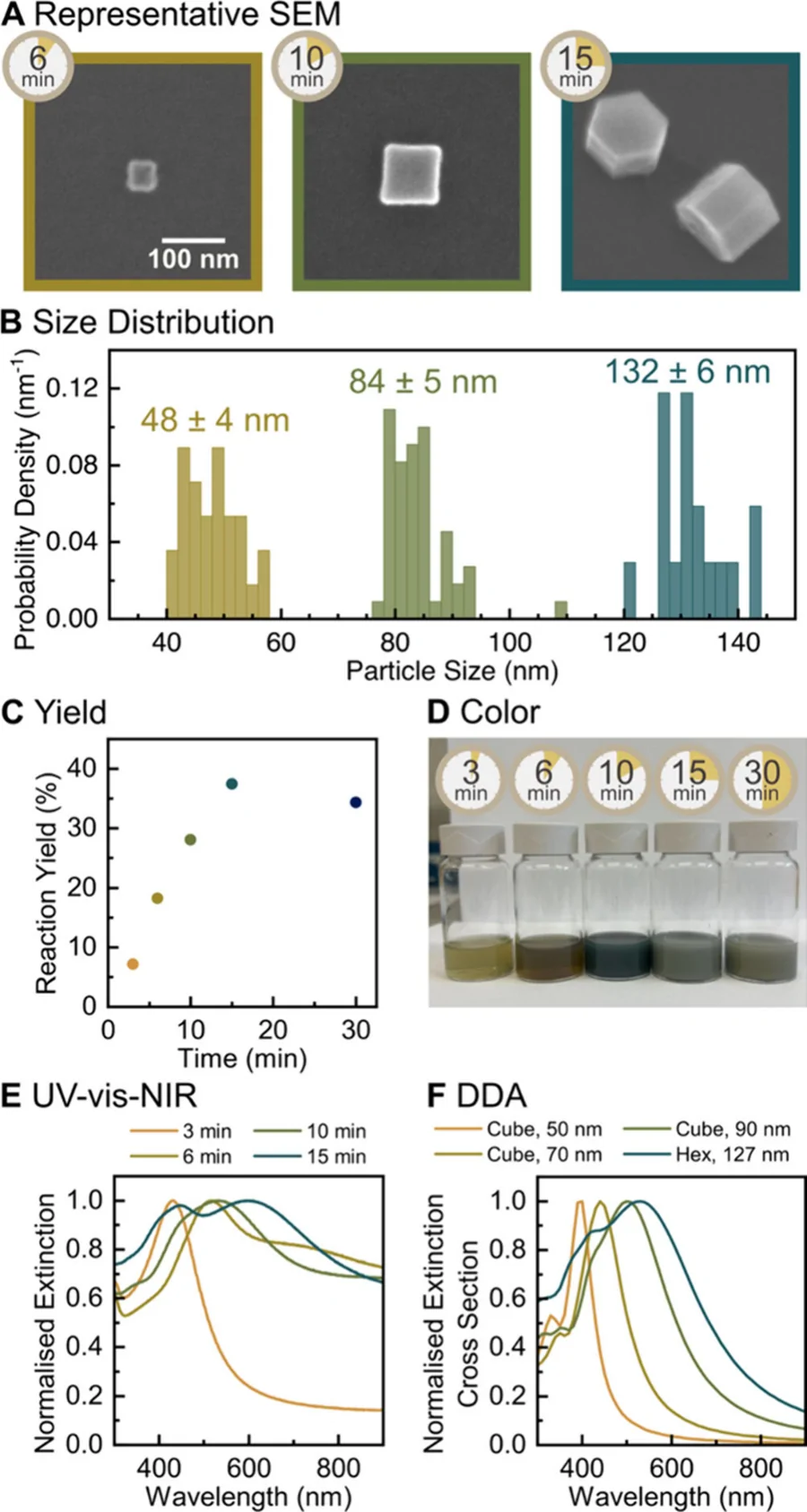
Mg nanocube growth. (A) Representative SEM (additional images in Figure S10), (B) NP size distribution measured face-to-face for nanocubes and tip-to-tip for hexagonal prisms, shown on the same plot for all three samples, (C) reaction yield of aliquots from the same reaction at different reaction times, (D) color photographs of product solutions, (E) experimental extinction spectra, and (F) numerical extinction spectra (DDA) matching the experimental shape and average NP size, including a conformal 10 nm oxide layer.

No nanocubes were detected at 15 and 30 min: hexagonal prisms were instead obtained, their tip-to-tip size was 132 ± 6 nm at 15 min (N = 17, Figure 2A,B, Figure S10). Mg reduction is continuous until 15 min of reaction (7%, 18%, and 28% yield at 3, 6, and 10 min), then plateaus, within experimental error, with yields of 37% at 15 min and 34% at 30 min (Figure 2C). Thin Mg hexagonal platelets of over 400 nm tip-to-tip appeared as a minority product (<5% particle count) in all samples, in agreement with previous results.^16^

The colloids formed at different reaction stages had starkly different colors ranging from yellow to red, dark blue, and finally gray (Figure 2D). Correspondingly, the extinction spectrum broadens and red-shifts for the growing Mg nanocubes, and two extinction maxima are present for the hexagonal prisms, assigned to the dipole and quadrupole modes (Figure 2E). The experimental extinction spectra match well the calculated profiles obtained via electromagnetic simulations for optical excitation in the discrete dipole approximation (DDA, Figure 2F, details in the Supporting Information).^21,22^ The low energy peak for the nanocubes arises from a corner mode while the weaker higher energy peaks, barely discernible in the experimental spectra but clear in the numerical results, can be tentatively assigned to previously reported^23,24^ edge and face modes. However, experimental spectra display a large, flat near-infrared absorption that is absent from the numerical results. This feature is attributed to the optical response of the hexagonal platelets in the samples (numerical simulations reported in Figure S11).

The bulk extinction feature attributed to the minority population of hexagonal platelets disappeared when the plasmonic response of nanocubes was interrogated at the single particle level, with dark field spectroscopy correlated with SEM. The scattering spectra of the larger Mg nanocubes (obtained after 10 min of reaction) exhibit one dominant dipole resonance within the experimentally probed wavelength range (representative spectra in Figure 3A, all others reported in Figure S12). Correlated SEM images support the narrow size distribution reported in Figure 2: the observed nanocube’s average size and standard deviation was 82 and 4 nm, respectively. The average resonance wavelength obtained was 491 nm (2.52 eV) with a full width at half-maximum (fwhm) of 122 nm. The resonance position (on glass in air) agrees well with the bulk extinction measurements performed in isopropanol—these two environments likely have comparable effective refractive indices for a cube sitting face-to-substrate.^25,26^ The narrow standard deviation on the extinction maxima (14 nm) and fwhm (17 nm) indicate not only a narrow size distribution but also a consistent shape and level of oxidation. Further, correlated optical scattering and SEM reveals that cube dimers can couple despite the presence of the oxide layer (Figure S13).

**Figure 3:**
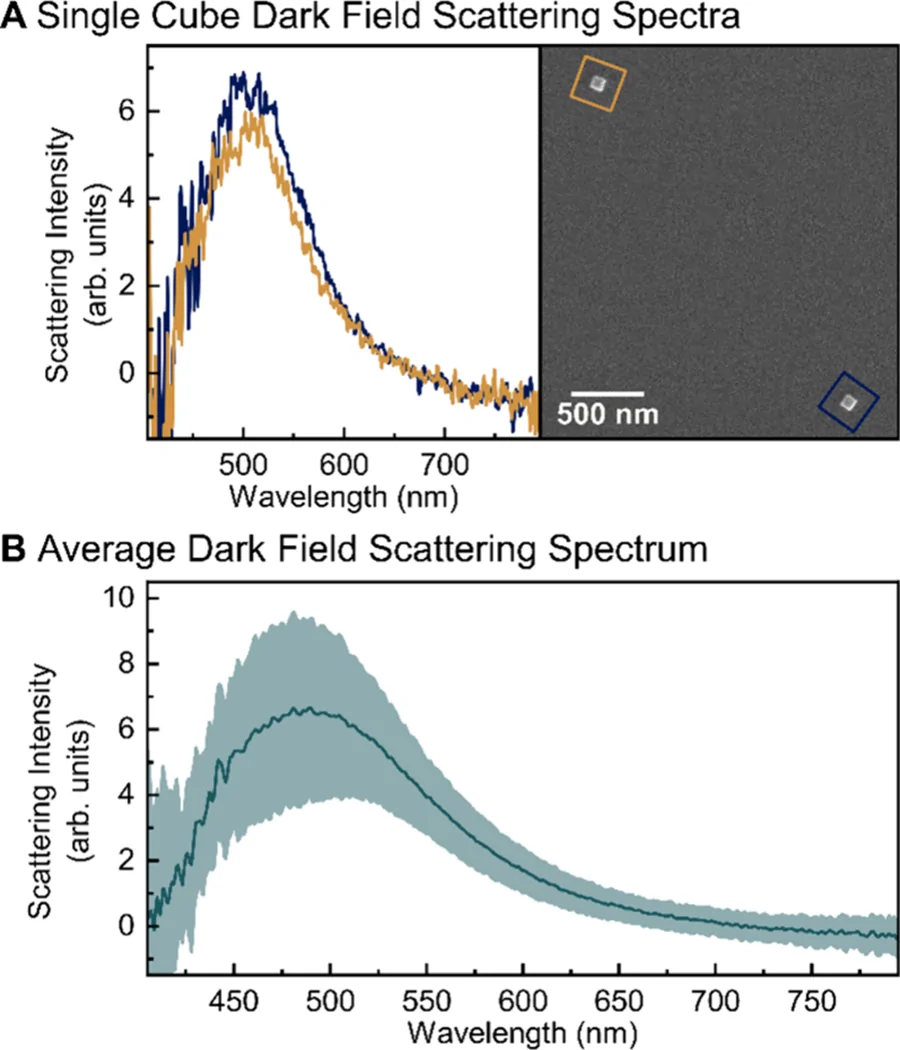
Dark field scattering from single Mg nanocubes. (A) Representative dark field scattering spectra and corresponding SEM images for two Mg nanocubes, and (B) average dark field scattering spectra and standard deviation obtained from 30 nanocubes (SEM and single particle scattering for individual particles reported in Figure S12).

To characterize the nanocube’s near-field response and confirm resonance mode assignment, plasmon excitation probability maps were obtained via monochromated electron energy loss spectroscopy in STEM (STEM-EELS). These reveal the local symmetry and spatial distribution of the plasmon modes at different energies as shown in Figure 4A for a 90 nm Mg nanocube (with five additional nanocubes reported in Figure S14). Measurements reveal three distinct EEL probability distributions: a corner mode (2.2 eV, 560 nm), a more localized, higher energy corner mode (2.8 eV, 440 nm) and an edge mode (3.5 eV, 350 nm). Numerical simulations of electron beam excitation (Figure 4B, Figure S15), indicate that, in the presence of the substrate, the dipole corner LSPR slightly red-shifts and separates from the other higher-order corner LSPRs. Field distributions obtained numerically for optical excitation (Figure S16) show that the substrate-LSPR coupling causes the field of the dipole (low energy) corner mode to localize more at the corners closer to the substrate, as opposed to the higher-order corner mode where the field appears stronger at the distal corners. This behavior is akin that reported by optical,^23,27,28^ numerical^23,27,28^ and electron microscopy^24^ studies of Ag nanocubes, where the lower energy corner mode is called proximal and the higher, distal. The presence of a MgO layer on Mg nanocubes likely weakens their coupling to the substrate when compared to Ag nanocubes coated with a thin polymer shell; numerical results indicate that when the MgO is removed a much stronger substrate interaction is observed, as expected (Figure S16).

**Figure 4:**
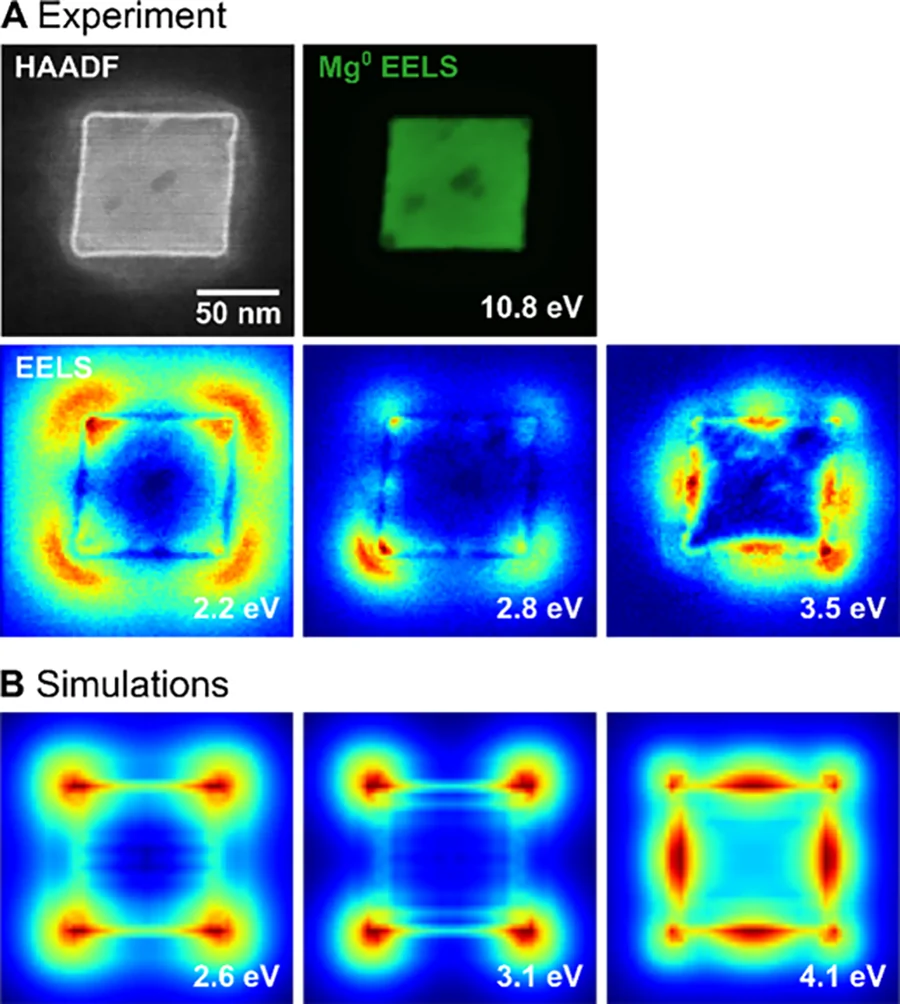
Near-field response of Mg nanocubes. (A) top: HAADF-STEM image and STEM-EELS Mg bulk plasmon map for a 90 nm side length nanocube, bottom: normalized spatial loadings of non-negative matrix factorization (NMF) components centered at the energy shown (spectral components reported in Figure S14A), and (B) simulated plasmon excitation probability maps for a 90 nm nanocube.

Higher in energy, an edge mode appears, matching well with the simulations. However, for this mode as well as the corner modes, the calculated peak energies are systematically too high. While the numerical model was as well-matched as possible to the experimental setup, including oxide layer and substrate, differences in the mode energy may be due to differences in the refractive index of the MgO (density or presence of hydroxide), overestimation of the MgO thickness, or the unexplained presence of small voids in the structures as visible in Figure 4. Finally, as expected from far-field observations, thick hexagons also exhibit a strong plasmonic response in the near field (Figure S17).

The synthesized Mg NPs crystallize in a hexagonal close-packed structure (HCP, space group *P*6_3_/*mmc*), as demonstrated by multiple single-particle electron diffraction patterns for nanocubes as well as for thick and thin hexagonal prisms, corroborated by corresponding simulated diffraction patterns (Figure 5, Figures S18–S21). The pattern symmetry and calculated lattice constants *a* and *c* for the various shapes (Table S1) match those of HCP Mg.^29^

**Figure 5:**
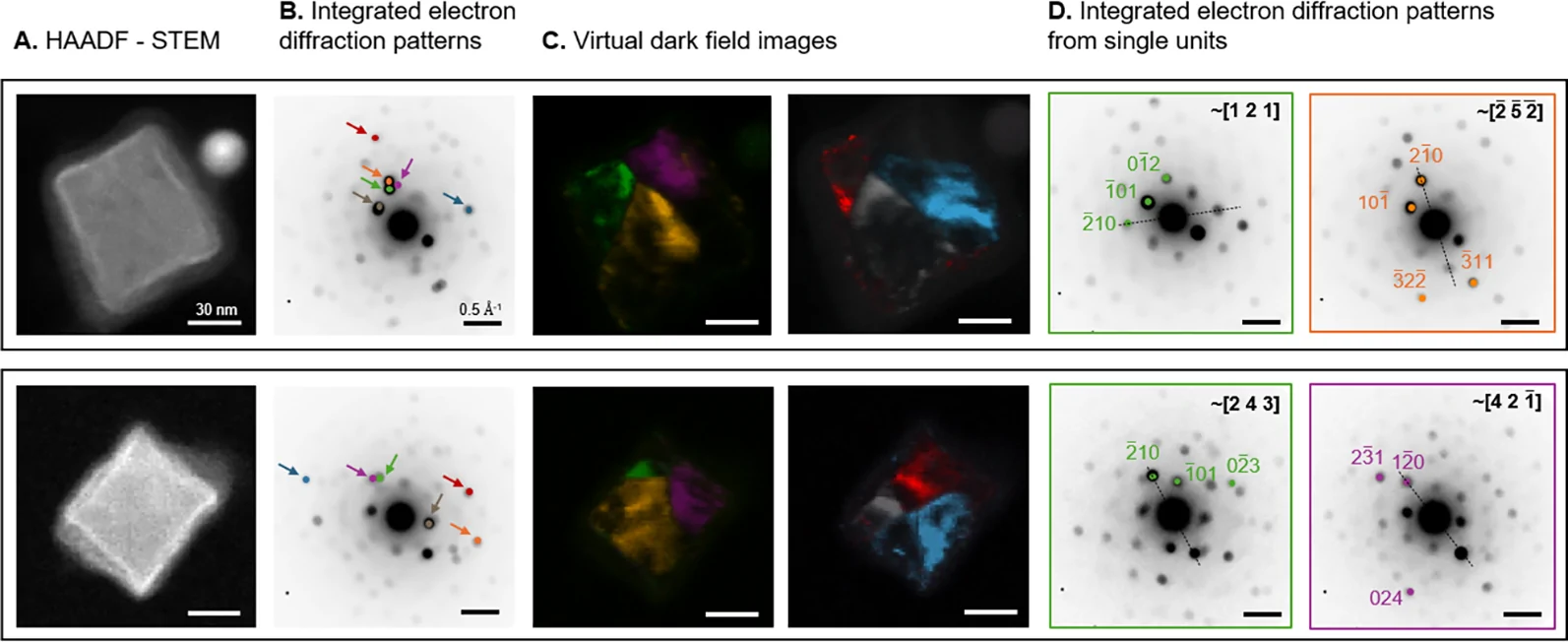
4D-STEM shows multiple twinning in Mg nanocubes with average side length of ∼76 nm (top) and ∼67 nm (bottom). (A) HAADF-STEM images (the circular feature on the top is carbon contamination), (B) integrated electron diffraction patterns, (C) virtual dark field images color-coded to corresponding diffraction points in (B), (D) integrated electron diffraction patterns from color-coded single crystal units. Virtual dark field image intensities adjusted for improved visualization. Dashed lines in (D) indicate the ⟨100⟩ axis. Scalebars: 30 nm for all HAADF-STEM and 0.5 Å^–1^ for all diffraction patterns.

Crystallographically, a cubic shape does not conform to the hexagonal symmetry of an HCP lattice: there does not exist six perpendicular facets of the same type in an HCP crystal. Therefore, Mg nanocubes are unlikely to be single crystalline. To verify this hypothesis, we performed 4D-STEM on multiple nanocubes, acquiring a diffraction pattern at each pixel of an image, yielding a 4D data set. Placing a virtual aperture on a diffraction point enabled the creation of a virtual dark field image, i.e., an image revealing areas producing the same diffraction point, oftentimes the same crystallographic orientation. Virtual dark field images for a ∼76 nm nanocube revealed six distinct, single crystal units with four well-indexable patterns viewed roughly along the [121], [2̅5̅2̅], [142], and [432̅] crystallographic directions (Figure 5, Figure S20). A second cube (∼67 nm) viewed across three different tilt angles confirmed the assignment of six single crystal units (Figure 5, Figures S21–S22). Further non-negative matrix factorization (NMF) analysis, a statistical approach using minimal user input, produced the same single crystal unit assignments (Figures S23–S24), confirming the multiply twinned makeup of Mg nanocubes.

Diffraction patterns obtained for cubes sitting roughly flat on the substrate correspond to viewing directions very close to ⟨100⟩ and ⟨121⟩ (Figure 5, Figure S19). The [100] direction is normal to the (21̅0) prismatic plane, while the ⟨121⟩ directions are not normal to low-index crystallographic planes. This could explain the appearance of both flat and slightly concave/rough faces in some of the cubes as lower index planes are favored. In an HCP unit cell, the ⟨100⟩ and ⟨121⟩ crystallographic direction families can form two distinct angles, one of which is 90.0° such as in the pairs [100]/[121] and [100]/[121̅]. Additionally, for Mg, [121] and [121̅] are almost perpendicular (86.3°), creating the orthogonal scaffold needed to form a cube corner. The relative directions, and the formation of a six-facet structure, then becomes evident when the directions’ poles are plotted on a stereographic projection, shown in Figure S25 for all the 4 indexable patterns of the 76 nm cube. At this point we do not have a substantial understanding of the transition from cube to thick hexagons nor the latter’s crystallography.

The plethora of twin planes in Mg (7 observed in nanocrystals^15^) which can form mirror twin planes but also double twinning,^30,31^ makes the twin plane assignment challenging. In the 76 nm cube, we could identify (1̅13) as the twin plane between the orange and green single crystal units, and (11̅1) between the orange and blue single crystal units.

Lastly, the oxide layer on Mg nanocubes, formed postsynthetically,^32^ renders them resistant to further oxidation, consistent with previous reports on colloidally synthesized NPs of Mg.^10,14,33^ Samples were dried in air for TEM and optical spectroscopy and stored in isopropanol for over a year, all without visible changes in morphology, plasmonic properties, or diffraction signature.

In conclusion, the unexpected cubic shape of metallic Mg, produced in high yield in syntheses containing nucleating agents, can be explained by multiple twinning. The cubes are protected by a thin, self-limiting oxide layer and display multiple plasmon resonances, which have been investigated at the bulk and single-particle level. These results expand the set of shapes possible in HCP crystals, with an impact for controlling nanoengineered shape-dependent properties.

## Supplementary Material



## Notes

Experimental details for synthesis, microscopy, spectroscopy, and numerical simulations. Additional electron micrographs including SEM, high resolution, and tilt series, extinction spectra, diffraction patterns, optical scattering spectra correlated with electron micrographs, simulation results for optical (scattering, extinction, EELS) and diffraction responses, electron spectroscopy data, non-negative matrix factorization analysis of diffraction data, stereograms (PDF)
