# Supplementary material for: Squaring the Hexagon: Multiply Twinned Plasmonic Magnesium Nanocubes
Source: Nano Lett. 2026 Oct 19;26(43):15140–5. doi: 10.1021/acs.nanolett.6c04201 (PMC13750483; doi:10.1021/acs.nanolett.6c04201)
Supplement: Supplementary file 1 [file nl6c04201_si_001.pdf]

Supporting Information for

# Squaring the Hexagon: Multiply Twinned Plasmonic Magnesium Nanocubes


Christina Boukouvala,[1,2] Andrey Ten,[1] Vladimir Lomonosov*,[1] Emilie Ringe*[1,3]

1. Department of Materials Science and Metallurgy, University of Cambridge, 27 Charles Babbage Road, Cambridge CB3 0FS, United Kingdom
2. National Center for Electron Microscopy, Molecular Foundry, Lawrence Berkeley National Laboratory, Berkeley, California 94720, United States
3. Department of Earth Sciences, University of Cambridge, Downing Street, Cambridge CB2 3EQ, United Kingdom


## MATERIALS AND METHODS

**Materials**

Li pellets (99%), naphthalene (99%), polyvinylpyrrolidone (PVP, average molecular weight = 10,000), 1.0 M di-*n*-butyl magnesium ($MgBu_2$) in heptane, sodium bicarbonate ($NaHCO_3$, 99.7%), lithium carbonate ($Li_2CO_3$, 99%), anhydrous tetrahydrofuran (THF, 99.9%, containing 250 ppm of butylated hydroxytoluene inhibitor), and nitric acid (70%) were purchased from Sigma-Aldrich and used as supplied. Sodium sulfate ($Na_2SO_4$, 99.5%), sodium dodecyl sulfate (SDS, 99%), and acetone (99.8%, extra dry) were purchased from Fisher Scientific and used as supplied. Anhydrous isopropanol (IPA) and potassium bicarbonate ($KHCO_3$, 99%) were purchased from Fluorochem and used as supplied. All glassware were washed with nitric acid, rinsed with water, dried in a 110 °C oven, and flame-dried under vacuum before use.

**Synthesis**

In a typical Mg nanocubes synthesis, lithium naphthalene dianion ($Li_2$Napth) was first prepared by sonication (45 min using Allendale Ultrasonics Cavitek Professional 100 W, 3 L) of Li pellets (28 mg, 4.03 mmol) and dry naphthalene (256 mg, 2.00 mmol) in THF (10.75 mL), in the presence of the capping agent PVP (20 mg) and nucleating agent SDS (30 mg, 0.10 mmol), in a 25 mL Schlenk flask under Ar atmosphere at room temperature. $MgBu_2$ in heptane (1.75 mL of 1.0 M, 1.75 mmol) was injected quickly into the freshly-prepared $Li_2$Napth flask under stirring (550 rpm) at room temperature (**WARNING! $MgBu_2$ is pyrophoric and should be handled under inert conditions**). The reaction was left to stir (550 rpm) under Ar atmosphere for 10 min before it was quenched by the injection of acetone (2 mL). Intermediate products were obtained by collecting aliquots (0.8 mL) using a glass pipette under the Ar atmosphere of a glovebox and quenching the aliquot by adding it to 4 mL of 20% acetone in THF. The resulting solid product was recovered by centrifugation (8,000 rcf, 10 min). Residual byproducts were removed by centrifugation (10,000 rcf, 10 min) and redispersion cycles in THF twice, acetone, and then IPA before the product was finally redispersed in IPA of original volume.

$Na_2SO_4$ (30 mg, 0.21 mmol) or $NaHCO_3$ (20 mg, 0.24 mmol) were used in place of SDS to also produce Mg nanocubes. Mg nanospheroids were produced by decreasing the amount of SDS (15 mg, 0.052 mmol) or without SDS, while a bimodal distribution of thin hexagonal platelets and nanospheroids was produced by increasing the amount of SDS (60 mg, 0.21 mmol and 120 mg, 0.42 mmol). Coral-like Mg NPs were produced by using $KHCO_3$ (20 mg, 0.20 mmol) in place of SDS. No effect was observed

when adding $Li_2CO_3$ (20 mg, 0.27 mmol) in place of SDS. Hexagonal nanoprisms were produced at longer reaction times (15 and 30 min) with SDS (30 mg, 0.10 mmol).

**Bulk extinction**

Ultraviolet-visible-near infrared (UV-Vis-NIR) extinction spectroscopy was performed on Thermo Fisher Evolution 220 UV-visible spectrophotometer with Mg NPs suspended in IPA at room temperature, in polymethyl methacrylate (PMMA) semimicro cuvettes.

**Single particle dark field optical scattering**

Dark field optical scattering spectroscopy was performed as detailed in Kumar et al.[1] in an inverted Nikon microscope equipped with a Princeton Instruments IsoPlane SCT320 spectrometer, a ProEM HS 1024 × 1024 EMCCD camera, and a Physik Instrumente P-545.3C7 piezoelectric stage controller. A Nikon D-LH/LC halogen lamp was used to illuminate the sample, drop-cast Mg nanoparticles on no.1.5 glass coverslips, through a 0.95-0.80 numerical aperture Nikon dark field condenser; scattered light was collected via a 100x Nikon CFI Plan Fluor oil immersion objective set to <0.8 numerical aperture. The single particle spectra, corrected for the contributions of the halogen lamp profile, background and dark noise, were fitted with a Lorentzian function in MATLAB. After single particle scattering spectroscopy, the sample was carbon coated with an 8 nm conductive carbon film then imaged in a FEI Nova NanoSEM scanning electron microscope (SEM) instrument operated at 5 keV and equipped with an ET secondary electron detector.

**Experimental and analysis details for electron microscopy, diffraction, and spectroscopy**

Scanning electron microscopy images of samples drop-cast on silicon wafers, or carbon-coated samples on glass coverslips, were obtained on a FEI Nova NanoSEM or ZEISS GeminiSEM operated at 5 kV.

A Thermo Fisher Scientific Spectra 300 operated at 200 kV was used to collect, from samples drop-cast on ultrathin carbon films (Ted Pella), 4D-STEM diffraction maps via a Quantum Detectors' Merlin direct electron camera with 256 X 256 pixels (0.7 mrad convergence angle and a 91.1 mm camera length, except for Figure S14F, 146.1 mm camera length), STEM-EDS via a Bruker SuperX detector, and STEM-EEL spectra (bulk plasmon maps, hexagonal prism LSPR maps) using a monochromator and a Gatan Continuum spectrometer and detector.

A Thermo Fisher Scientific Krios operated at 200 kV in STEM was used to collect, from samples drop-cast on ultrathin carbon films (Ted Pella), the HAADF-STEM tilt series in Figure S8, using a camera length of 87 mm.

A Nion UltraSTEM 100MC High Energy Resolution Monochromated EELS STEM (HERMES) operated at 60 kV was used to collect STEM-EEL spectra (cube LSPR maps) of samples drop-cast on 20 nm thick $Si_3N_4$ films (SIMPore) using Nion's ultrahigh-resolution ground-potential monochromator, and a Nion Iris spectrometer with a Dectris ELA hybrid pixel detector camera. Corresponding high-angle annular dark field (HAADF) images were collected at an angular range of 90-195 mrad.

For the 4D-STEM diffraction maps, the python-based package pyXem[2] was used to align the diffraction pattern central beam and correct for annular distortion. Virtual dark field images were obtained by integrating the diffraction intensity within circular regions of interest centered on selected diffraction spots. Scikit-learn[3] was used for the non-negative matrix factorization (NMF). Prior to performing NMF, the central beam was masked and the diffraction patterns were background corrected via a radial median filter. The algorithm performed better after applying a square-root transform on the data, consistent with the Poissonian statistics expected from a direct electron camera.

For STEM-EDS spectral data, the python-based package eXSpy v0.3.1[4] was used to integrate X-ray line intensities for each element. The background windows for the $K_\alpha$ lines of Mg (1.25 keV) and O (0.52 keV) were set between the regions below and above the peaks in the summed spectra. A linear background connecting the windows below and above each peak was fit. The peaks were then integrated above the background to obtain elemental maps.

STEM-EEL spectral data from Nion UltraSTEM 100MC HERMES were aligned to sub-pixel accuracy, using the zero-loss peak (ZLP), and denoised using principal component analysis on the Gatan Digital Micrograph software. STEM-EELS spectral data from Thermo Fisher Scientific Spectra 300 were aligned to sub-pixel accuracy, using the ZLP, with the python-based package eXSpy v0.3.1.[4]
The resulting EEL spectral data from both microscopes were processed using the python-based package eXSpy v0.3.1.[4] Mg bulk plasmon maps were obtained by integrating the spectrum between 9.0 and 11.0 eV, to capture the bulk plasmon peak. LSPR modes were extracted using NMF in the region between between 0.60 and 8.00 eV (cube) and 0.80 and 9.00 eV (hexagon), after the spikes were removed by linear interpolation and minimum intensity was shifted to 0. A Lorentzian shape was fitted to NMF spectral factors to obtain component energies.

**Numerical simulations**
Single Crystal version 5.1.3 was used to perform electron diffraction simulations. All simulations were calculated for hexagonal close packed (HCP) Mg with a = 3.2093 Å and c = 5.2103 Å. The camera length was set to 91.1 mm, the electron beam energy to 200 keV, and the convergence angle to 0.7 mrads, to match experimental conditions. The spot size and sample thickness have little impact on the results of this kinematic diffraction simulation and were set to 0.06 $Å^{-1}$ and t = 20 Å.

Optical scattering spectra were obtained numerically in the discrete dipole approximation using DDSCAT version 7.3.2[5,6] while EELS calculations were performed using a modified version of eDDA,[7,8] a version of DDSCAT in turn modified to replace the plane wave stimulation with that of a swift electron beam. The frequency-dependent refractive index (RI) of metallic Mg was taken from Pehlivan et al.[9] and the RI of MgO and was set to 1.7. The ambient RI was set to 1.0 when modelling the response of NPs dried on a substrate and to 1.3772 for NPs dispersed in IPA. All calculations were carried out with a dipole distance of 2 nm, including a 10 nm MgO shell unless stated otherwise. In order to include the effect of the experimental TEM support film, the cube was placed on top of a 20 nm thick $Si_3N_4$ substrate with RI = 2.05 that extended 40 nm beyond the edges of the cube. EEL probability maps were calculated with a 4 nm energy step size.

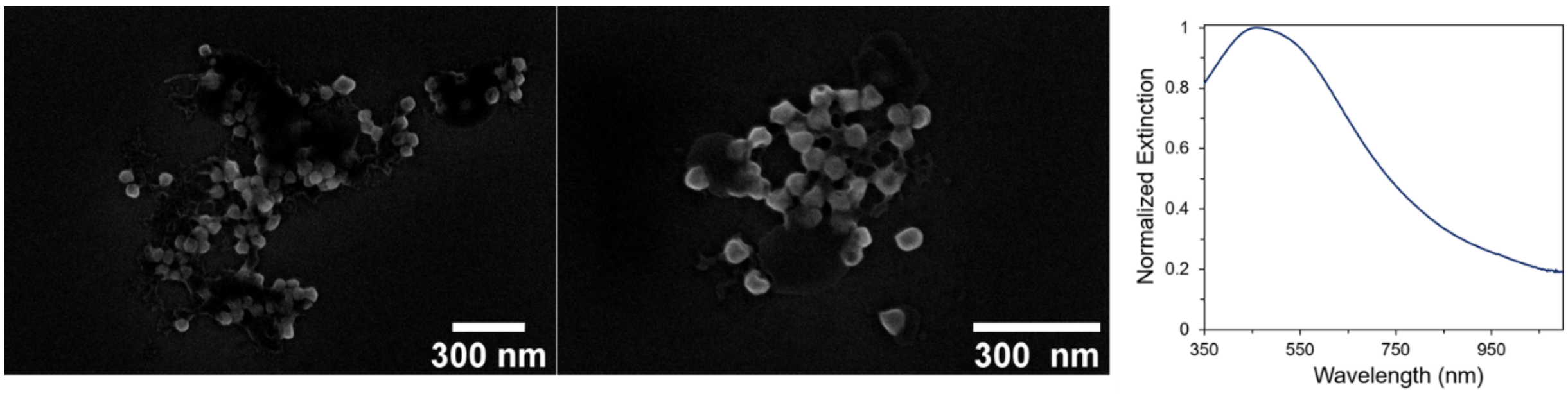


Figure S1. Left and center: SEM images of spheroidal Mg nanoparticles obtained without nucleating agent. Right: Bulk extinction of a suspension of spheroidal Mg nanoparticles in IPA.

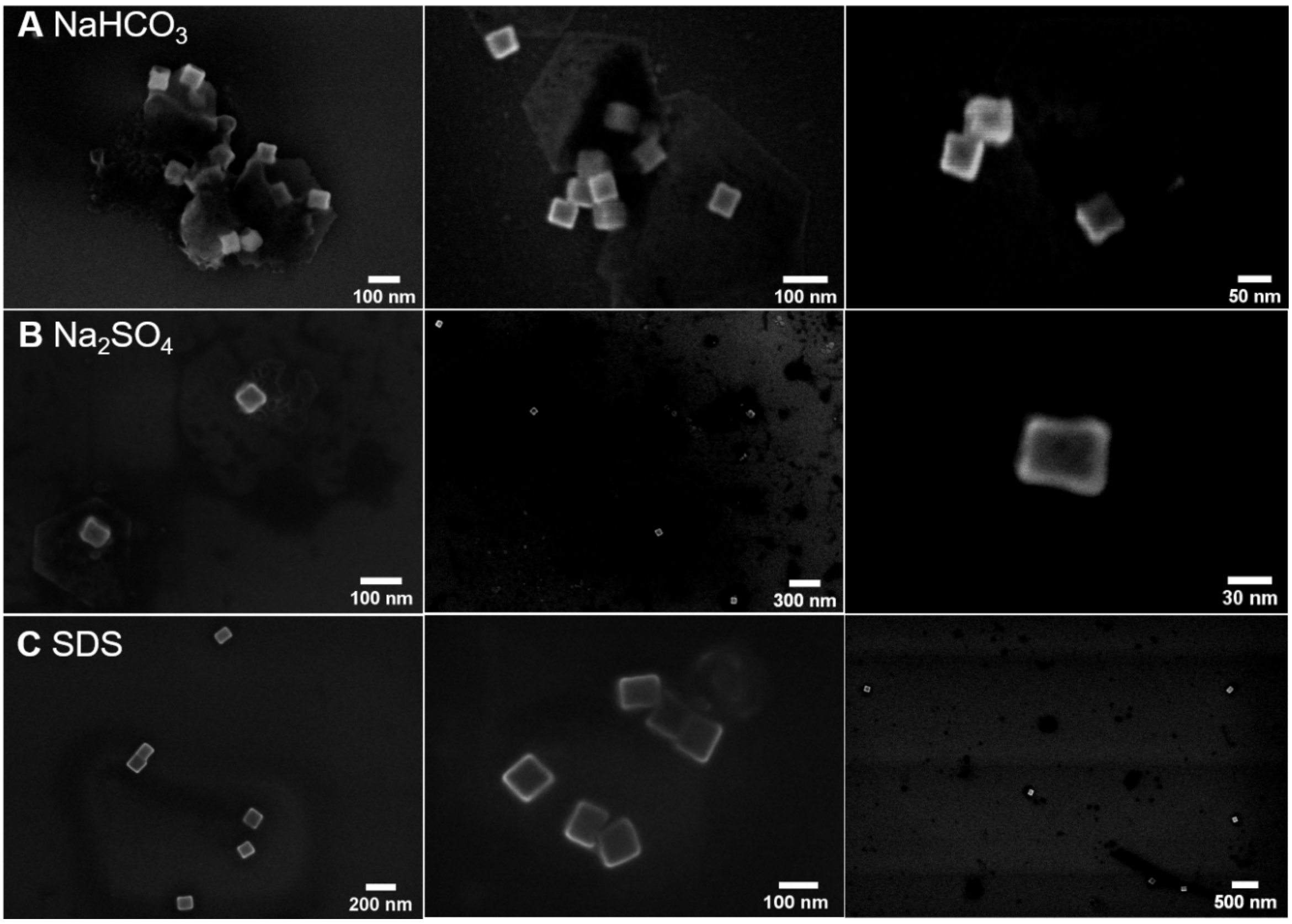


Figure S2. SEM images of Mg nanocubes obtained with different Na-containing nucleating agents: A) sodium bicarbonate (20 mg, 0.24 mmol), B) sodium sulfate (30 mg, 0.21 mmol), C) sodium dodecyl sulfate (SDS, 30 mg, 0.10 mmol).

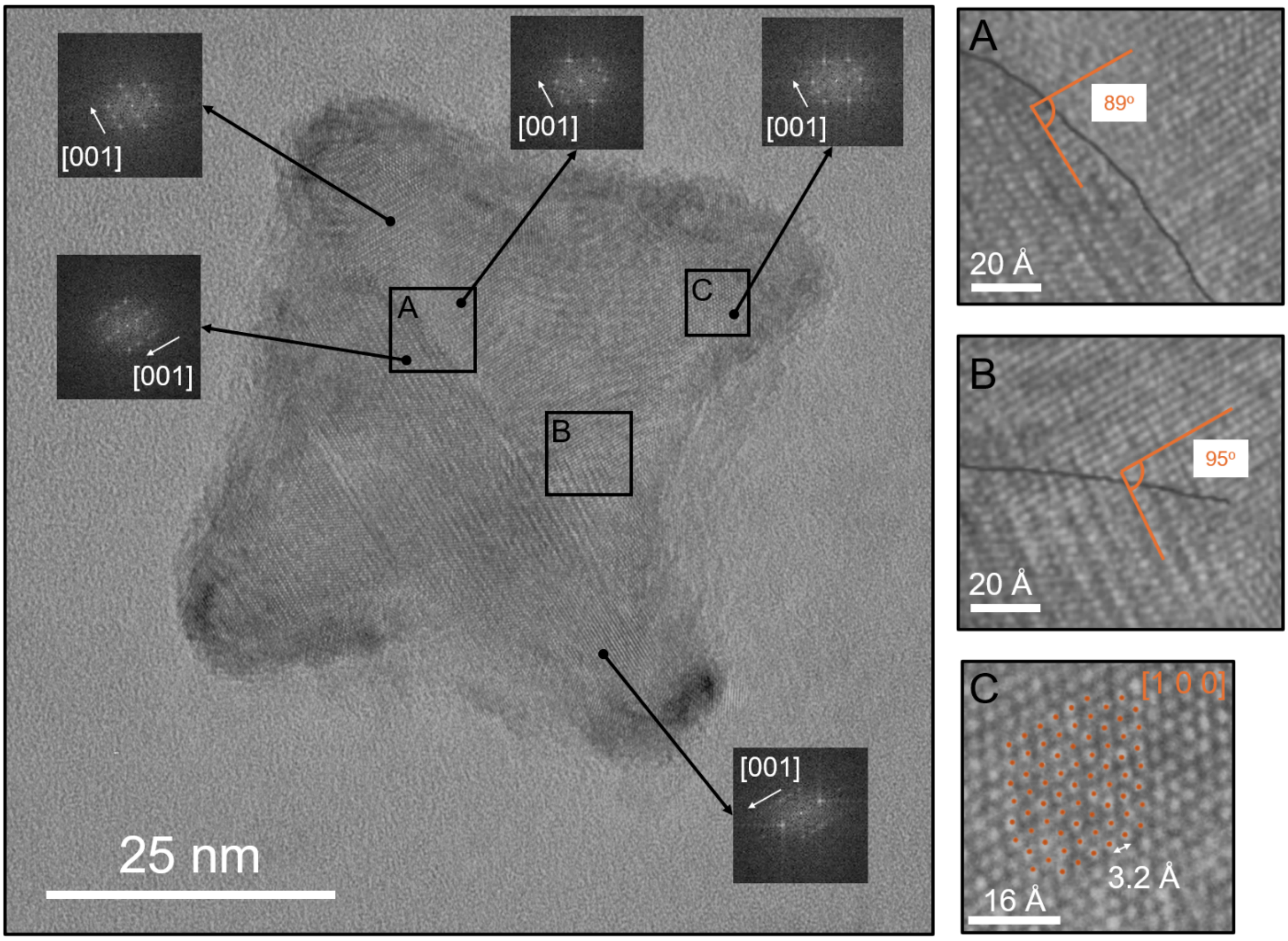


Figure S3. High resolution TEM of a Mg nanocube showing cubic shape and twinning. Fast Fourier Transform (FFT) patterns obtained at different regions of the HRTEM image reveal the lattice symmetry and orientation. A, B) Twinning boundaries. C) atomic columns overlaid with predicted positions (orange disks) viewed along the [100] zone axis.

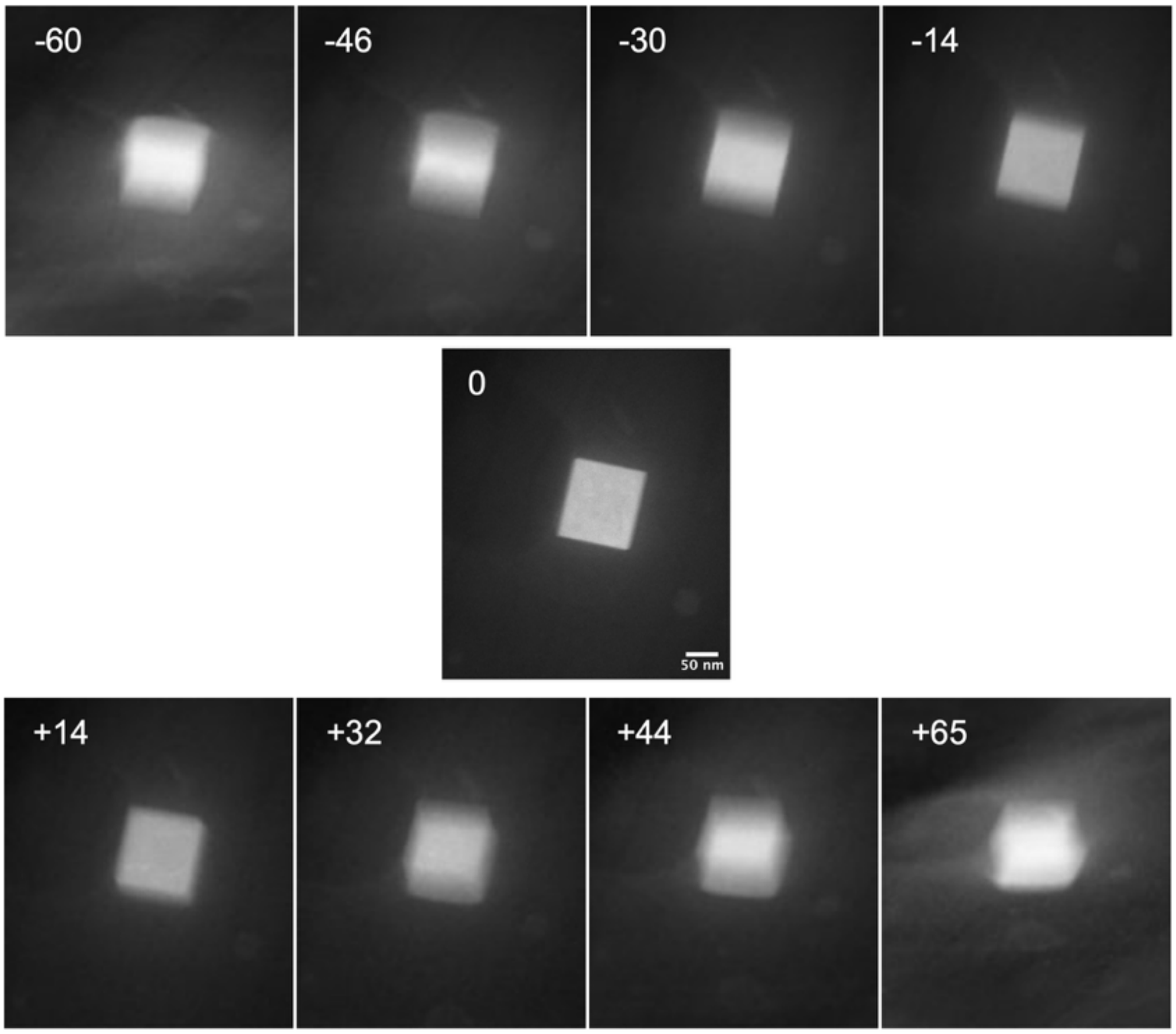


Figure S4. HAADF-STEM tilt series of a Mg nanocube. The scalebar applies to all images.

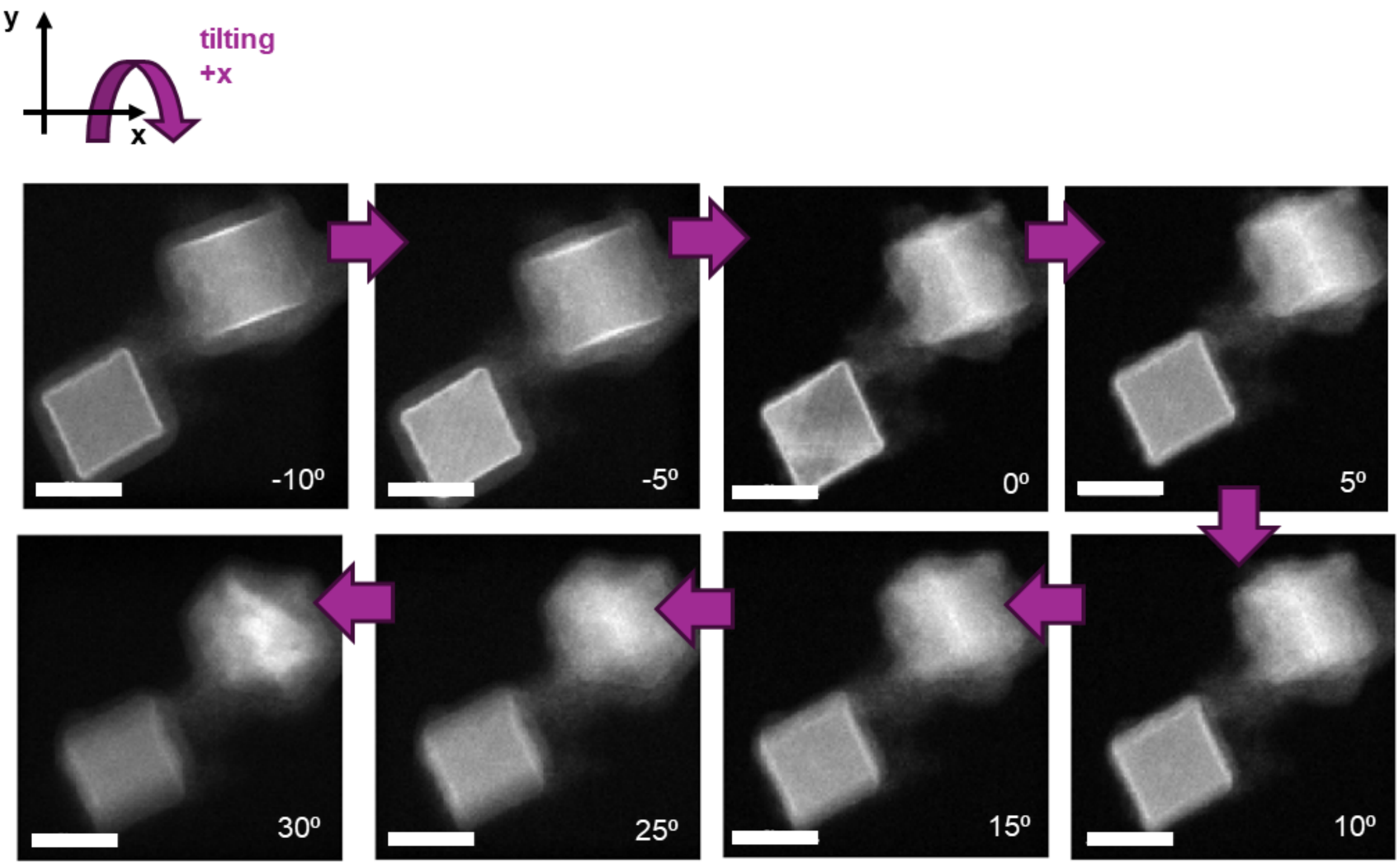


Figure S5. HAADF-STEM tilt series of two Mg nanocubes. Scalebar, 70 nm.

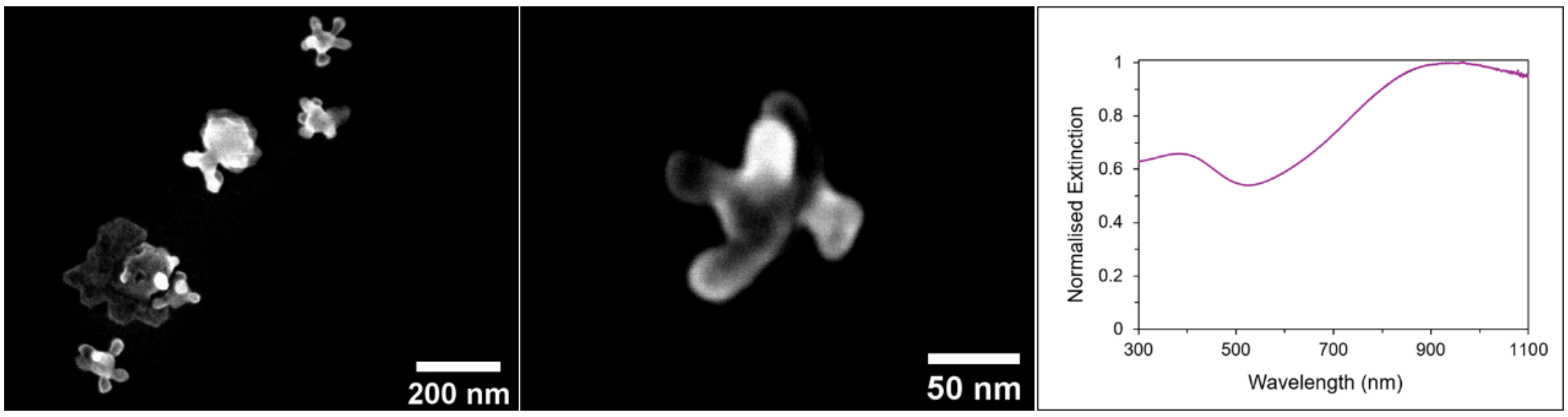


Figure S6. Left and center: SEM images of coral-like Mg nanostructures obtained in the presence of potassium bicarbonate (20 mg, 0.20 mmol). Right: Bulk extinction of a suspension of coral-like Mg nanoparticles in IPA.

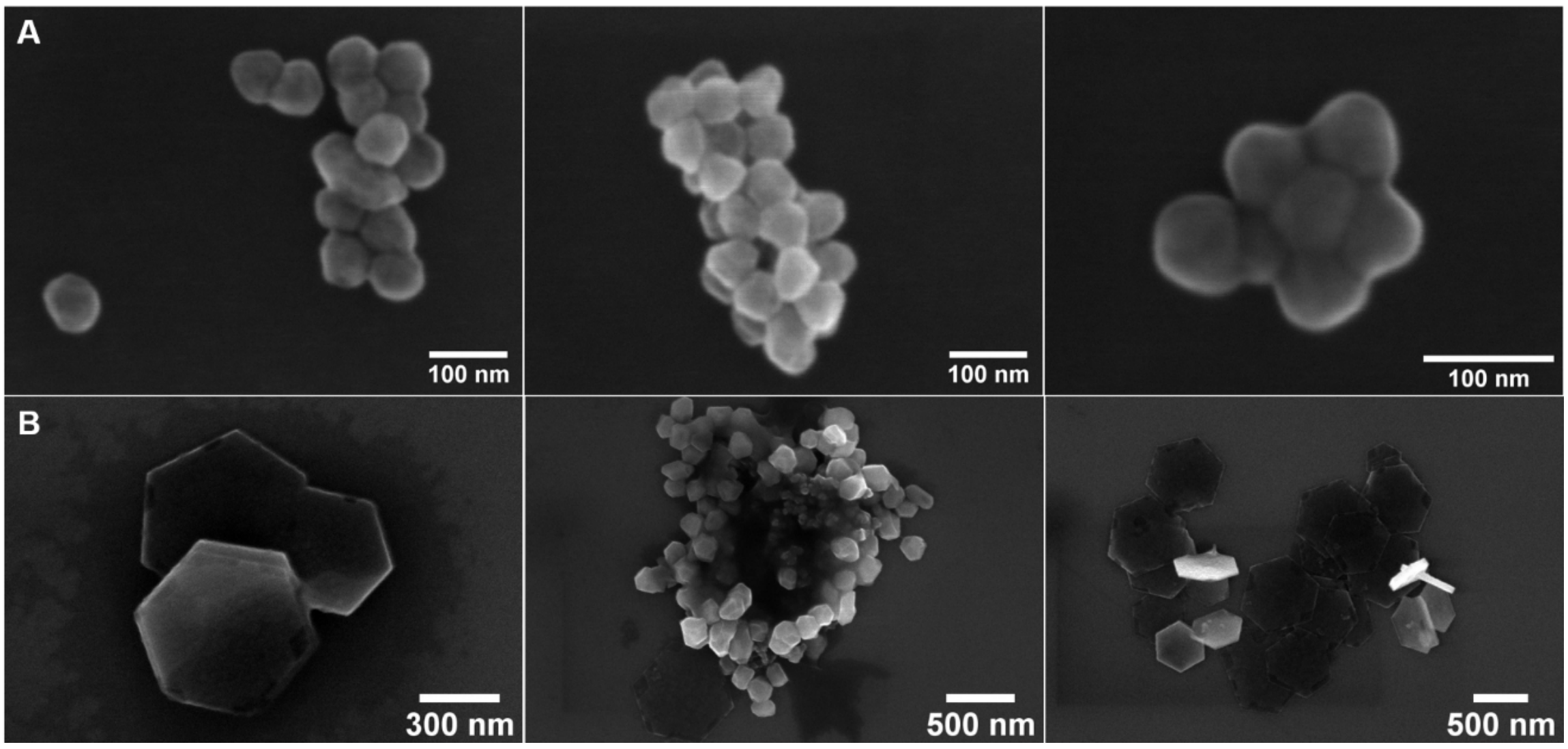


Figure S7. SEM images of representative Mg nanoparticles produced in the presence of A) 15 mg (0.052 mmol) of SDS (spheroidal NPs) and B) 60 mg (0.21 mmol) of SDS (bimodal sample composed of hexagonal platelets and spheroids; 120 mg SDS has the same morphology).

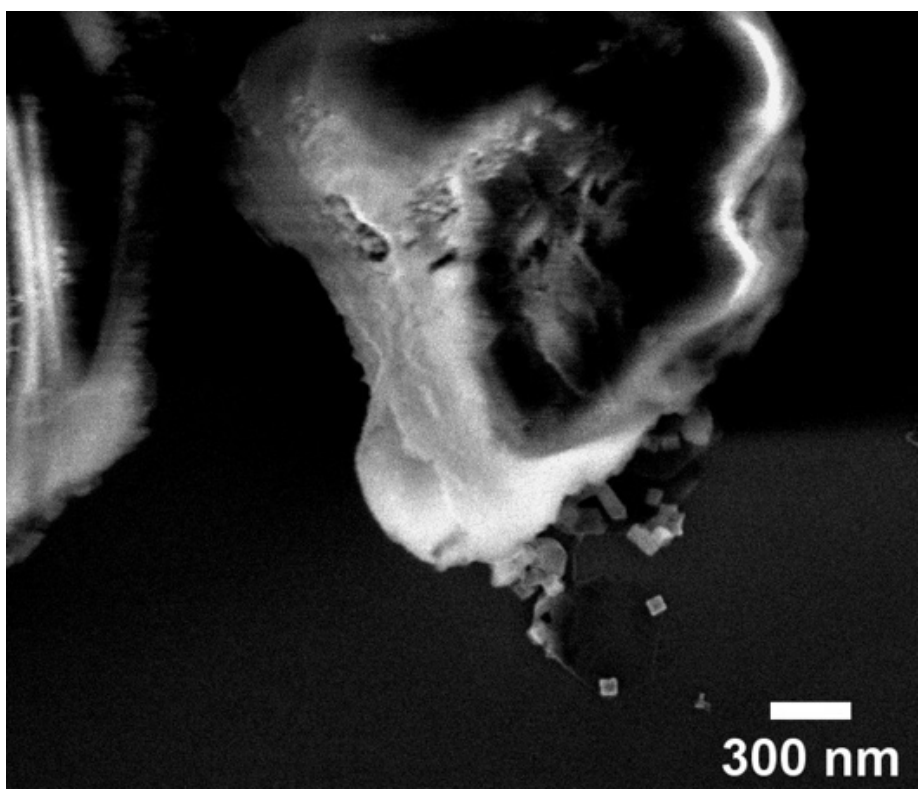


Figure S8. SEM image of large crystals of insoluble SDS contamination present in the nanocube samples.

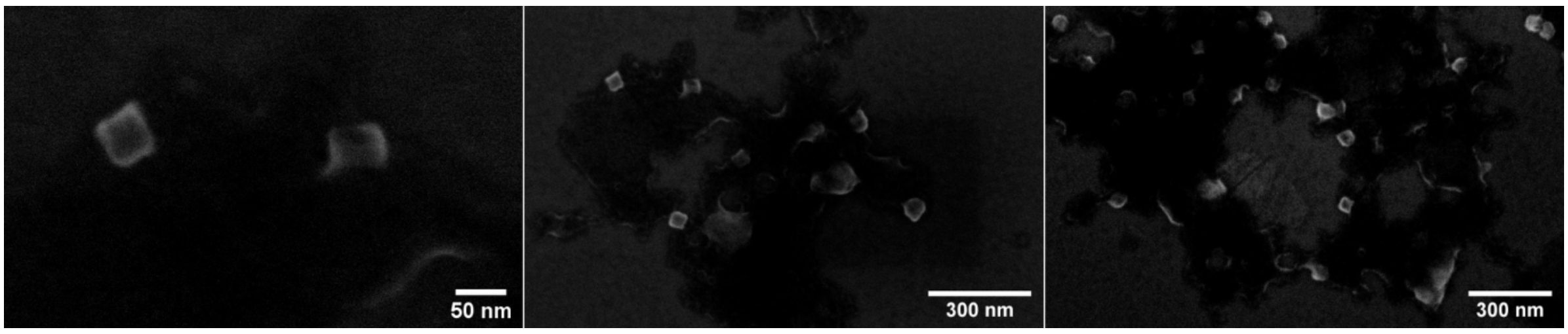


Figure S9. SEM images of sub-40 nm cube-like Mg nanoparticles formed after 3 min of reaction in the presence of SDS (30 mg, 0.10 mmol).

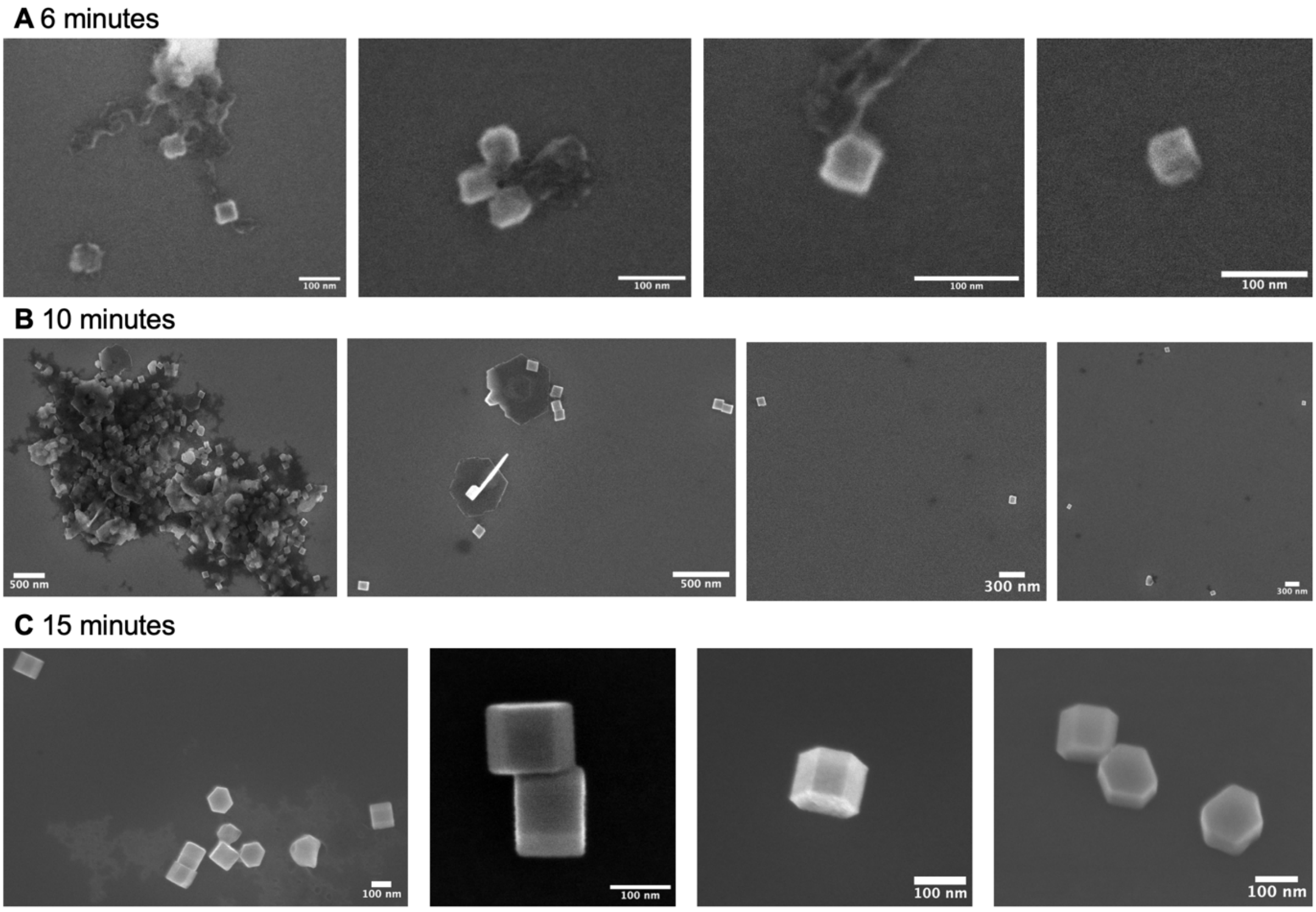


Figure S10. Additional SEM images of nanoparticles obtained from aliquots taken at 6, 10, and 15 minutes reported in Figure 2.

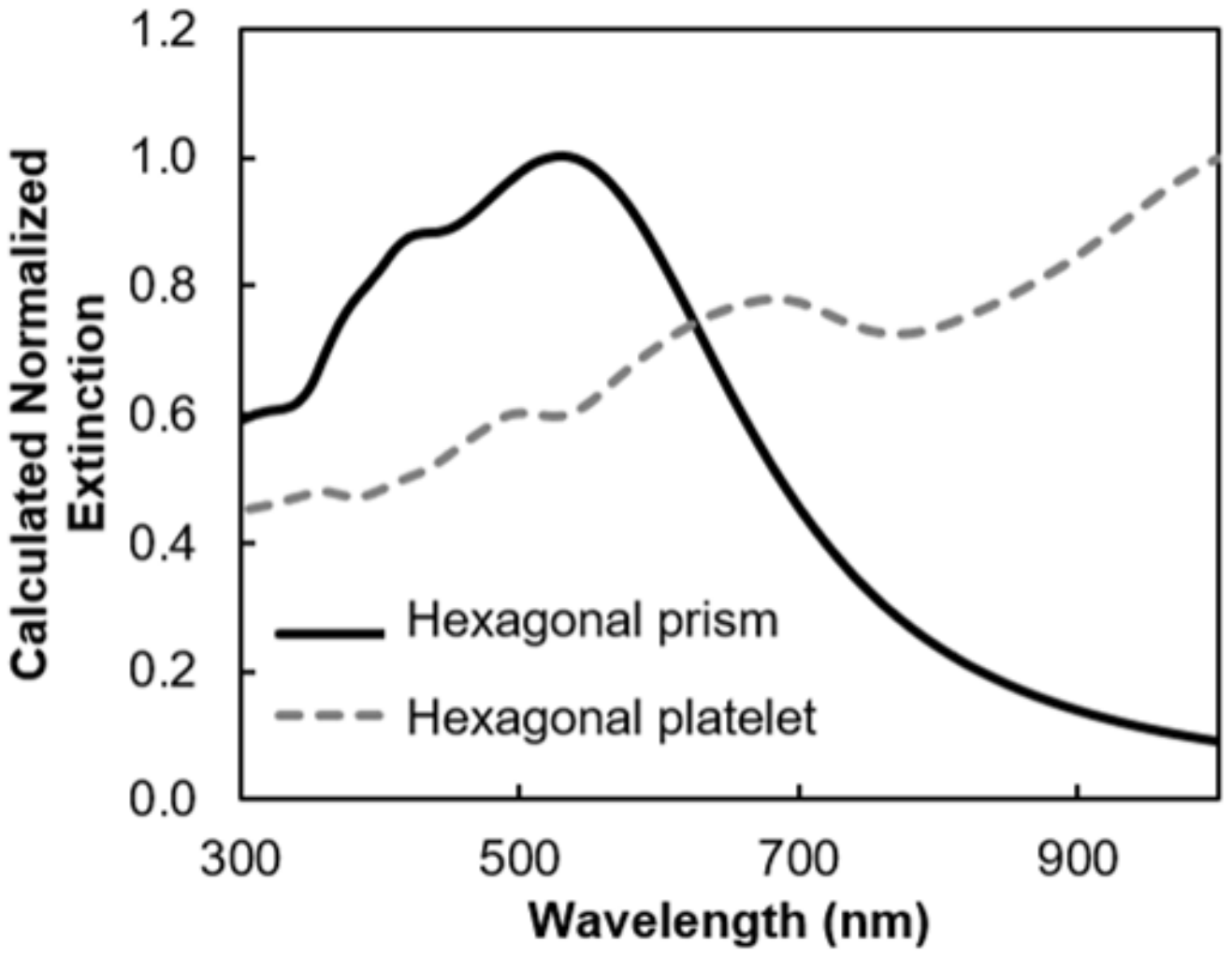


Figure S11. Simulated extinction spectra (in IPA) of a hexagonal prim (solid line) with a height of 124 nm and edge-to-edge length 110 nm (tip-to-tip 127 nm) and hexagonal platelet (dashed line) with thickness of 38 nm and edge-to-edge length of 422 nm. Reported sizes include a conformal 10 nm MgO shell.

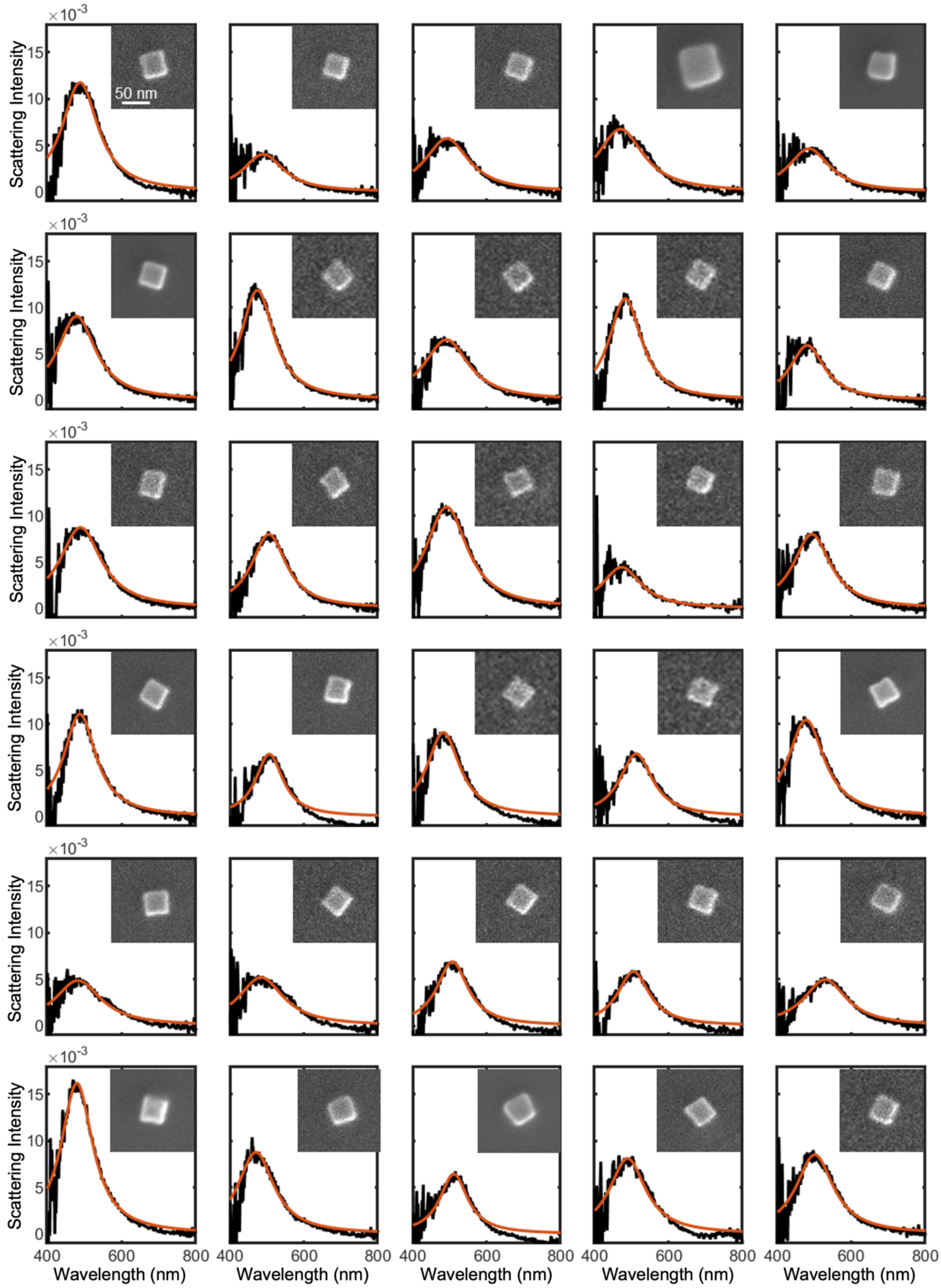


Figure S12. Single particle dark field scattering spectra (black line) with a Lorentzian fit (orange line) and insets with the corresponding SEM images for the 30 Mg nanocubes reported in Figure 4B.

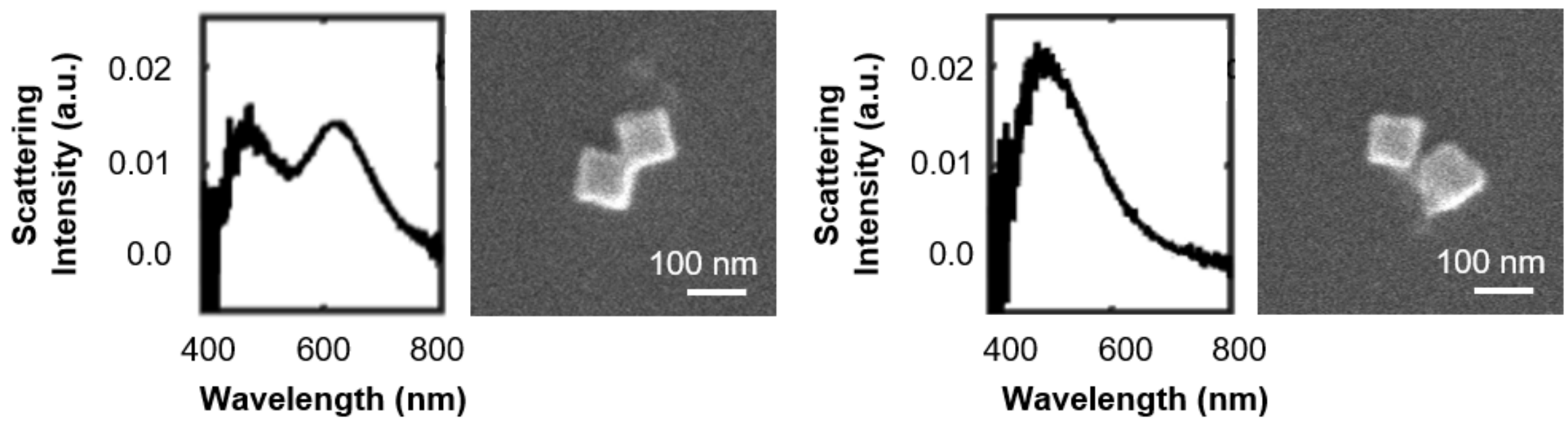


Figure S13. Single particle dark field scattering spectra with corresponding SEM images for coupled (left) and uncoupled (right) Mg nanocube dimers.

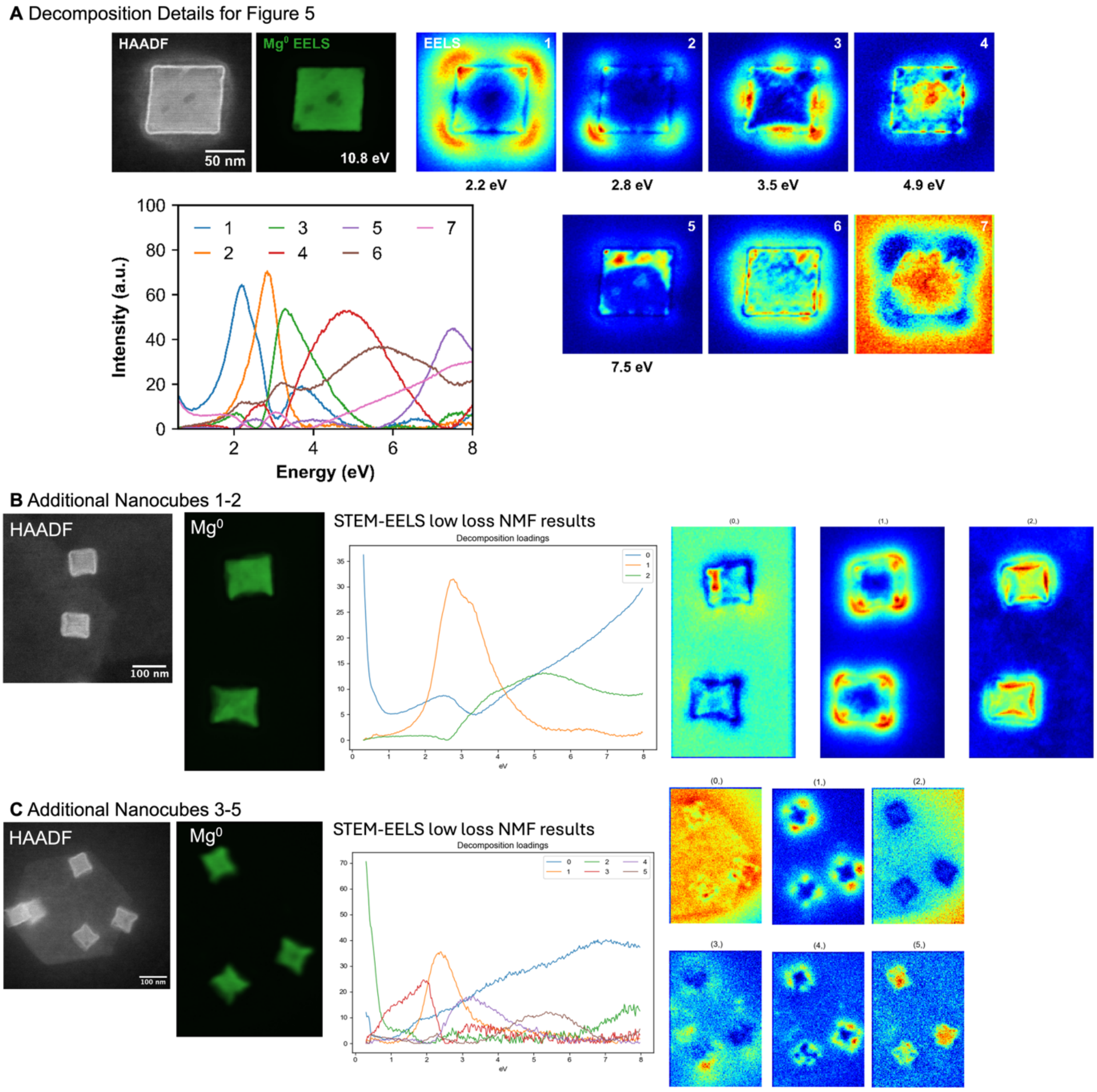


Figure S14. Details and additional STEM-EELS evidence of metallicity and plasmonic behavior of Mg nanocubes. A) Decomposition details for the nanocube shown in Figure 5. B-C) HAADF-STEM, bulk plasmon map and STEM-EELS NMF loadings (images) and factors (colored lines) for five additional nanocubes.

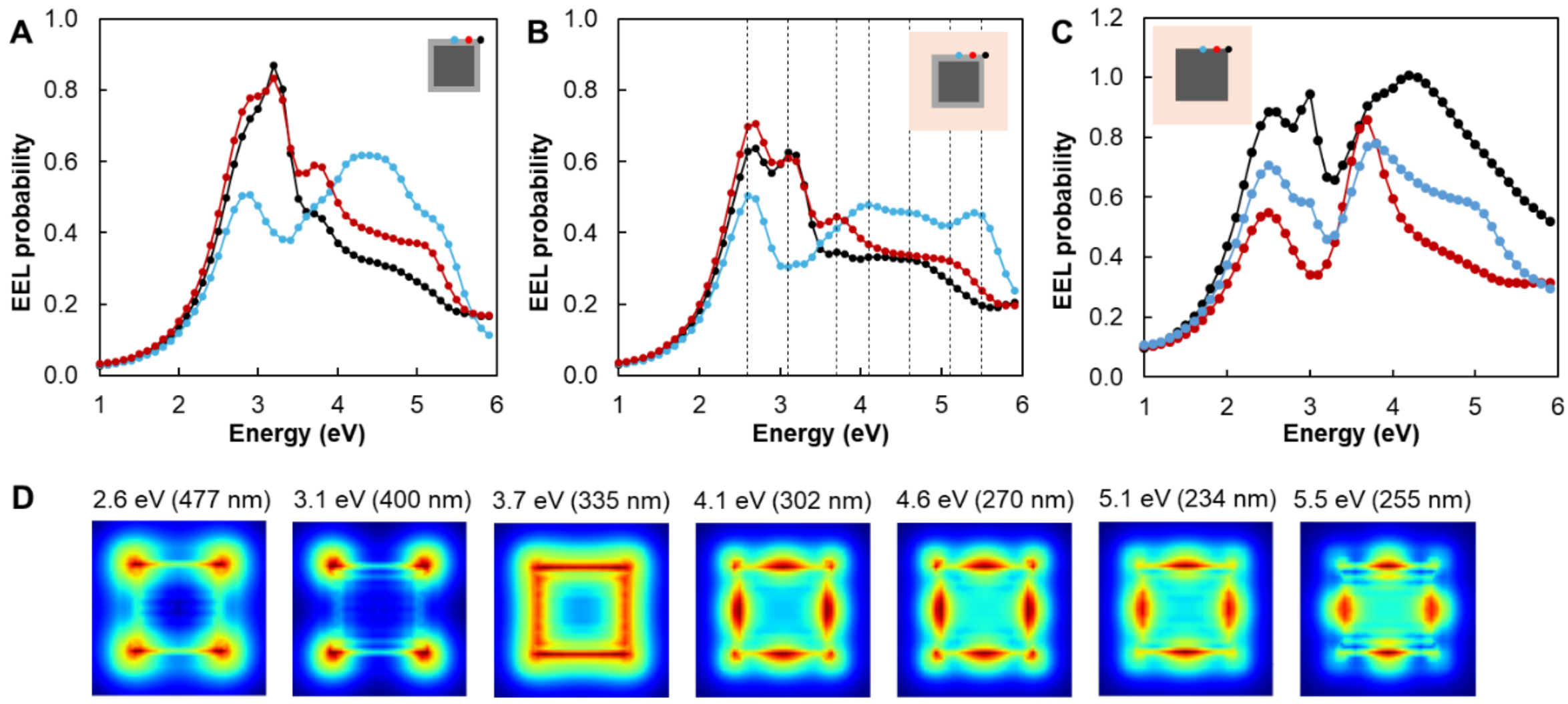


Figure S15. EELS simulations. Simulated EEL probability spectra for a Mg@MgO nanocube of side length, L, = 90 nm total including a 10 nm thick MgO shell, A) without and B) with a 20 nm thick $Si_3N_4$ substrate. Point spectra are shown at the tip (black), quarter edge length (red) and middle of the edge (blue) of the cube. C) EEL probability spectra for a Mg nanocube (L = 90 nm) without an MgO layer sitting on a 20 nm thick $Si_3N_4$ substrate. D) EEL probability maps corresponding to the dashed lines in B.

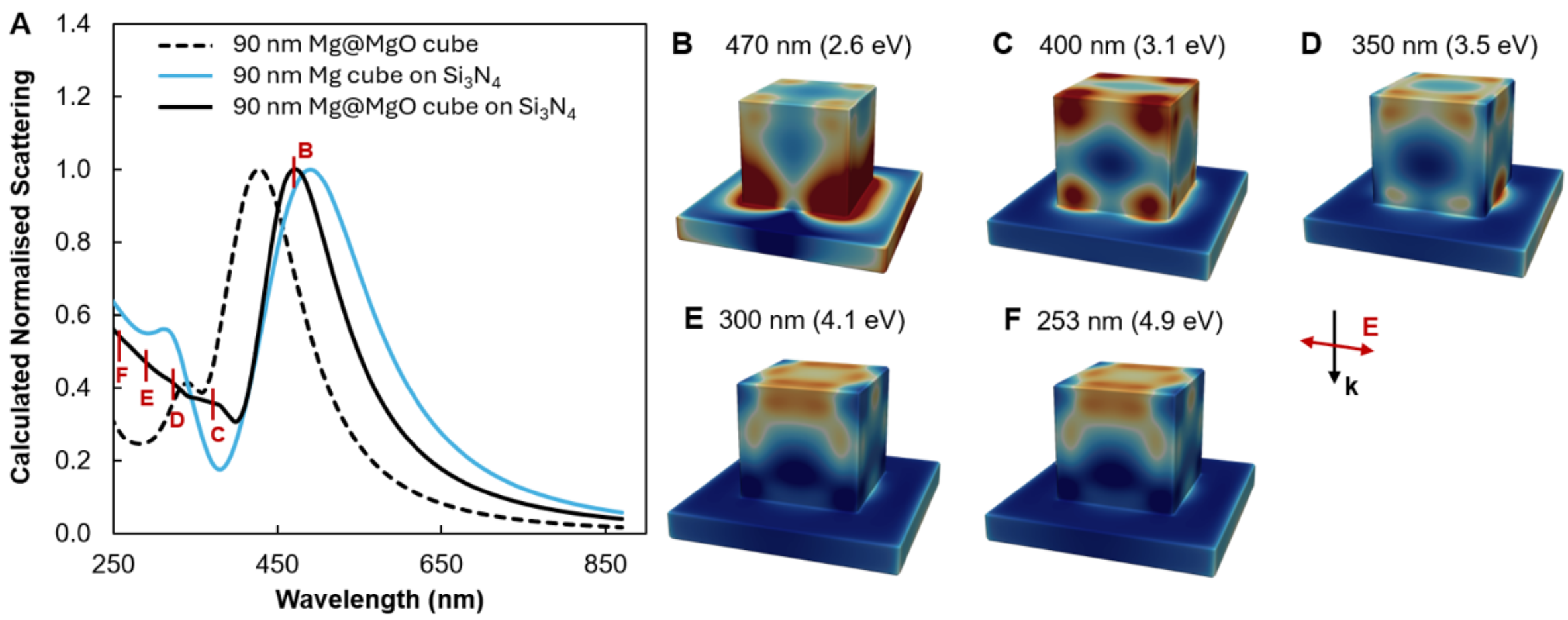


Figure S16. Optical excitation simulations. A) Calculated scattering for a Mg@MgO cube (L = 90 nm, $t_{MgO}$ = 10 nm) without a substrate (black dashed line), a Mg cube (L = 90 nm) on a $Si_3N_4$ substrate (solid blue line), and a Mg@MgO cube (L = 90 nm, $t_{MgO}$ = 10 nm) on a $Si_3N_4$ substrate (solid black line). B-E) Near-field enhancement contour, $|E|/|E_0|$, at different wavelengths corresponding to the red marks in A for the Mg@MgO cube on $Si_3N_4$. The refractive index of the surrounding environment was set to RI = 1.

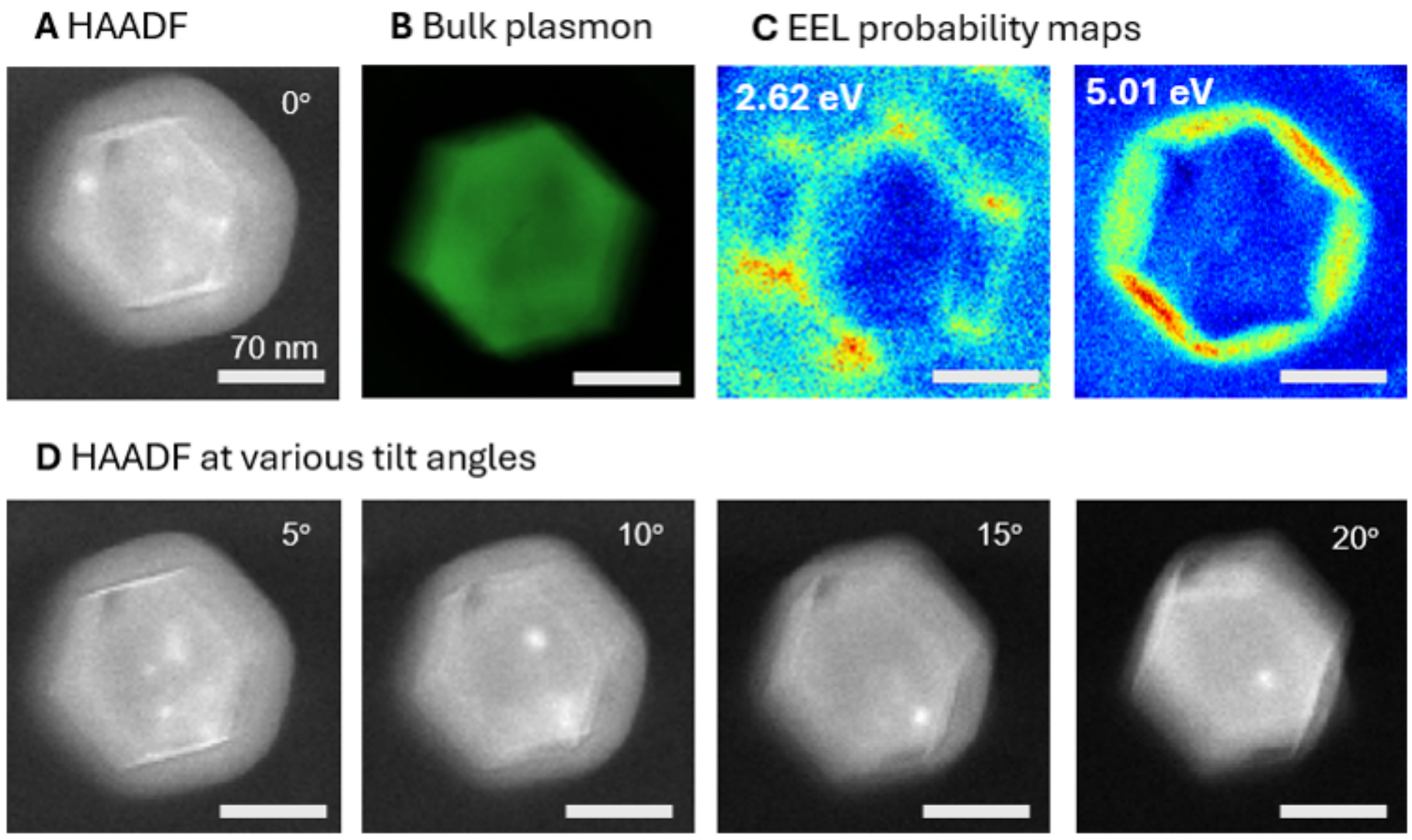


Figure S17. Shape and plasmonic response of a hexagonal prism. A) HAADF-STEM image, B) bulk plasmon resonance map, C) EELS NMF components centered at the energies shown, and D) HAADF-STEM tilt series.

Table 1. Lattice spacings (a and c for HCP Mg and a for FCC MgO) measured for different shapes shown in Figures S18 and S19. A least-squares fit of multiple measured diffraction spacings (n) was used to determine the lattice parameters in HCP Mg, along with the standard deviation (SD). The lattice constant c could not be measured for the hexagonal platelets as they preferentially sit with the basal plane (c axis) normal to the electron beam direction.

| lattice constants (Å) | a | SD | c | SD |
|---|---|---|---|---|
| **hexagonal platelet** (from Figure S18A, n = 3) | 3.217 | 0.065 | - | - |
| **hexagonal prism** (from Figure S18B, n = 5) | 3.213 | 0.022 | 4.934 | 0.180 |
| **cube** (from Figure S19K, n = 12) | 3.269 | 0.017 | 5.238 | 0.179 |
| **MgO** (from Figure S19J, n = 5) | 4.269 | 0.059 | | |

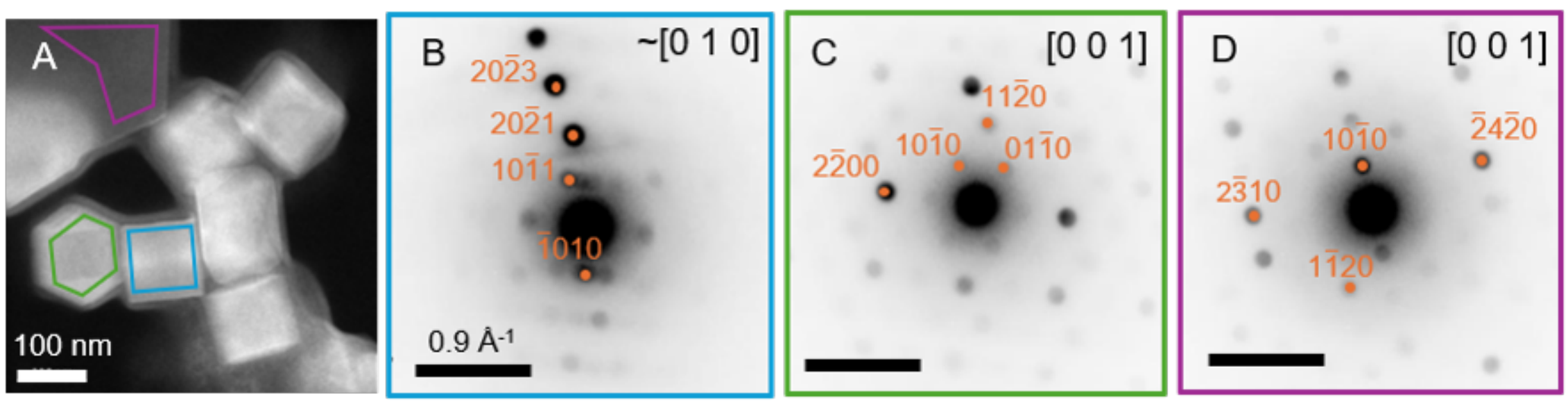


Figure S18. Electron diffraction patterns of hexagonal prisms and a hexagonal platelet. A) HAADF-STEM image. B-D) Integrated electron diffraction patterns over the corresponding color-coded regions in A for B) a hexagonal prism sitting on its edge (viewing direction [010]), C) a hexagonal prism sitting on its base (viewing direction [001]), and D) a hexagonal platelet sitting flat on the substrate (viewing direction [001]).

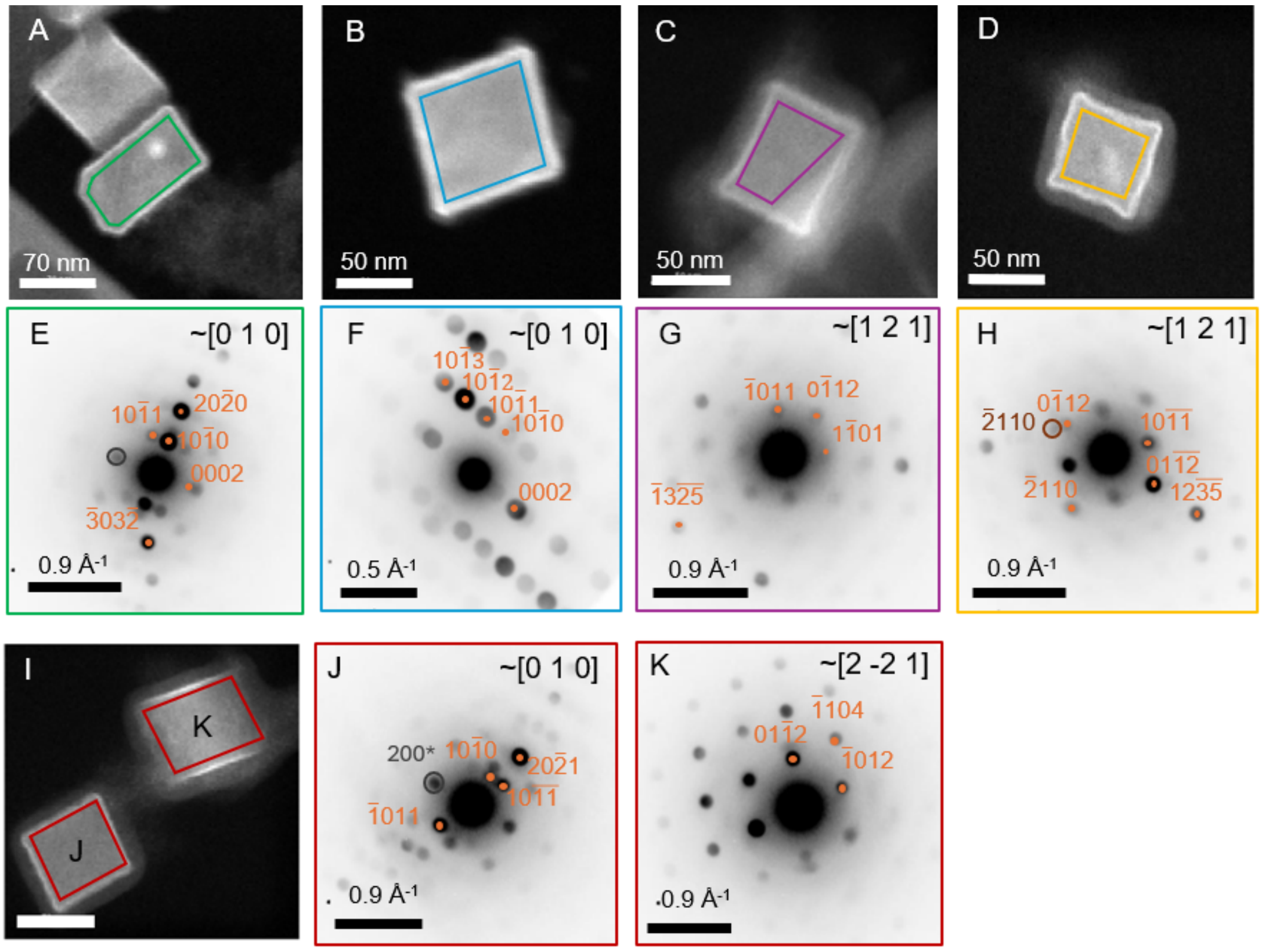


Figure S19. Diffraction pattens of various nanocubes. A-D and I) HAADF-STEM images. E-H and J-K) Diffraction patterns obtained by integrating the signal from the regions indicated with the color-coded boxes in A-D and I. The diffraction patterns in E, F, and J correspond to viewing directions close to [010] while those in G and H, close to [121]. The top cube in I is sitting roughly on its edge (see Figure S5, tilt series -10º) with a correspomding viewing direction close [2-11] as shown in K. The grey open circle in E and J corresponds to MgO's (200) plane while the brown open circle in H corresponds to the $(\bar{2}110)$ plane from a twinned crystal. Scalebar in I: 70 nm

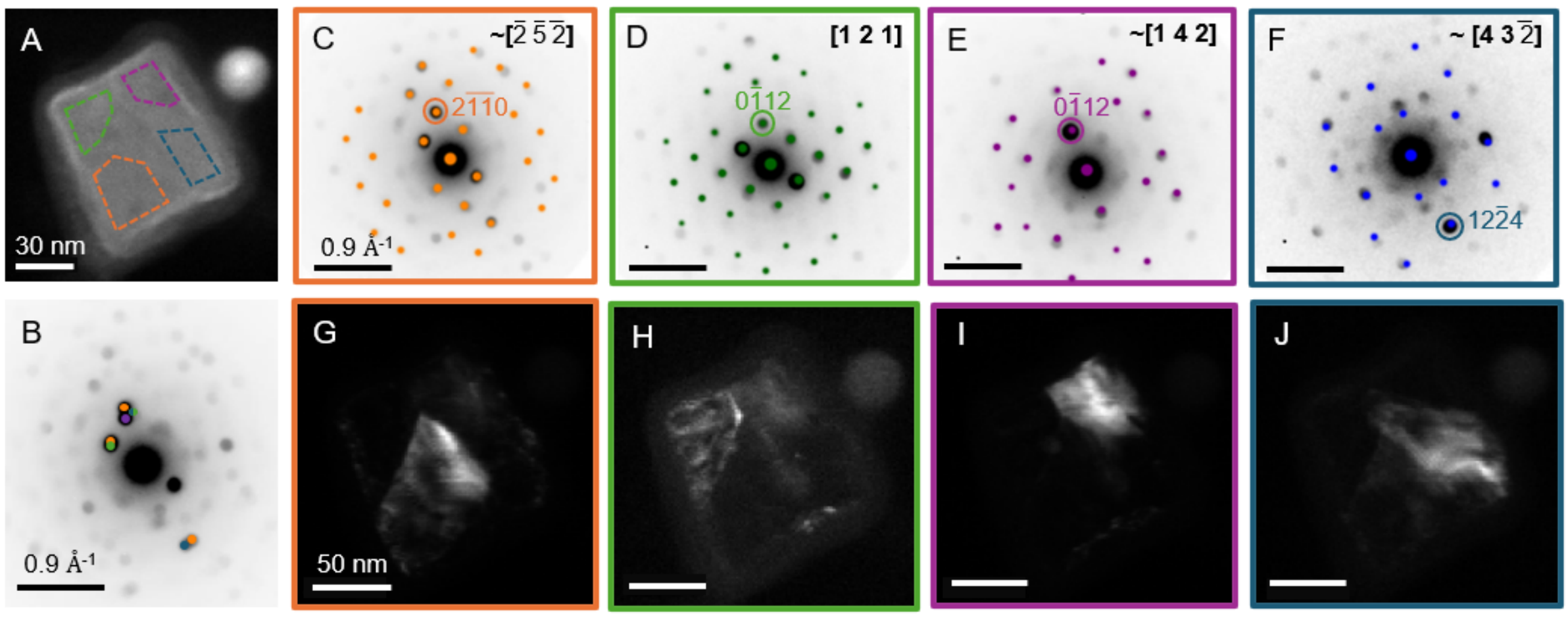


Figure S20. Multiply twinned Mg cube and main single crystal units. A) HAADF-STEM image and B) corresponding integrated diffraction pattern of a ~76 nm cube with some diffraction spots color-coded to the corresponding single crystal units. C-D) Diffraction patterns integrated over the regions of interest indicated with dotted lines in corresponding colors in A and overlaid with simulated diffraction patterns along the indicated orientations. G-H) Virtual dark field images corresponding to the circled diffraction points in C, D, E and F respectively.

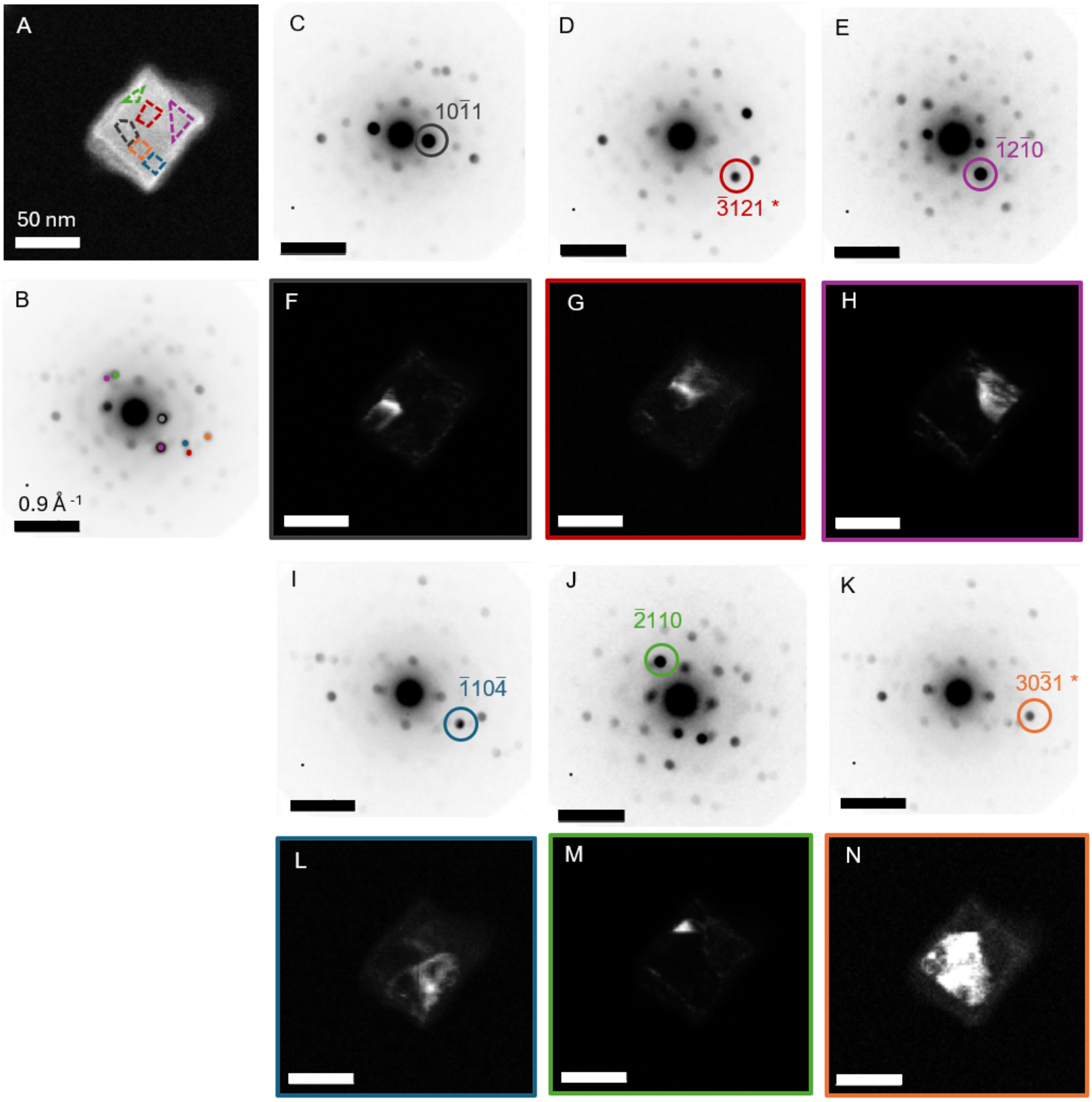


Figure S21. Multiply twinned Mg nanocube and its constituent single crystal units. A) HAADF-STEM image and B) corresponding integrated diffraction pattern of a ~67 nm cube with some diffraction spots color-coded to the corresponding single crystal unit. C-E) and I-K) Diffraction patterns integrated over the regions of interest indicated with dotted lines in corresponding colors in A. F-H) and L-N) Virtual dark field images corresponding to the circled diffraction points in the respective diffraction patterns. Diffraction spots indicated with a start (*) are tentatively assigned.

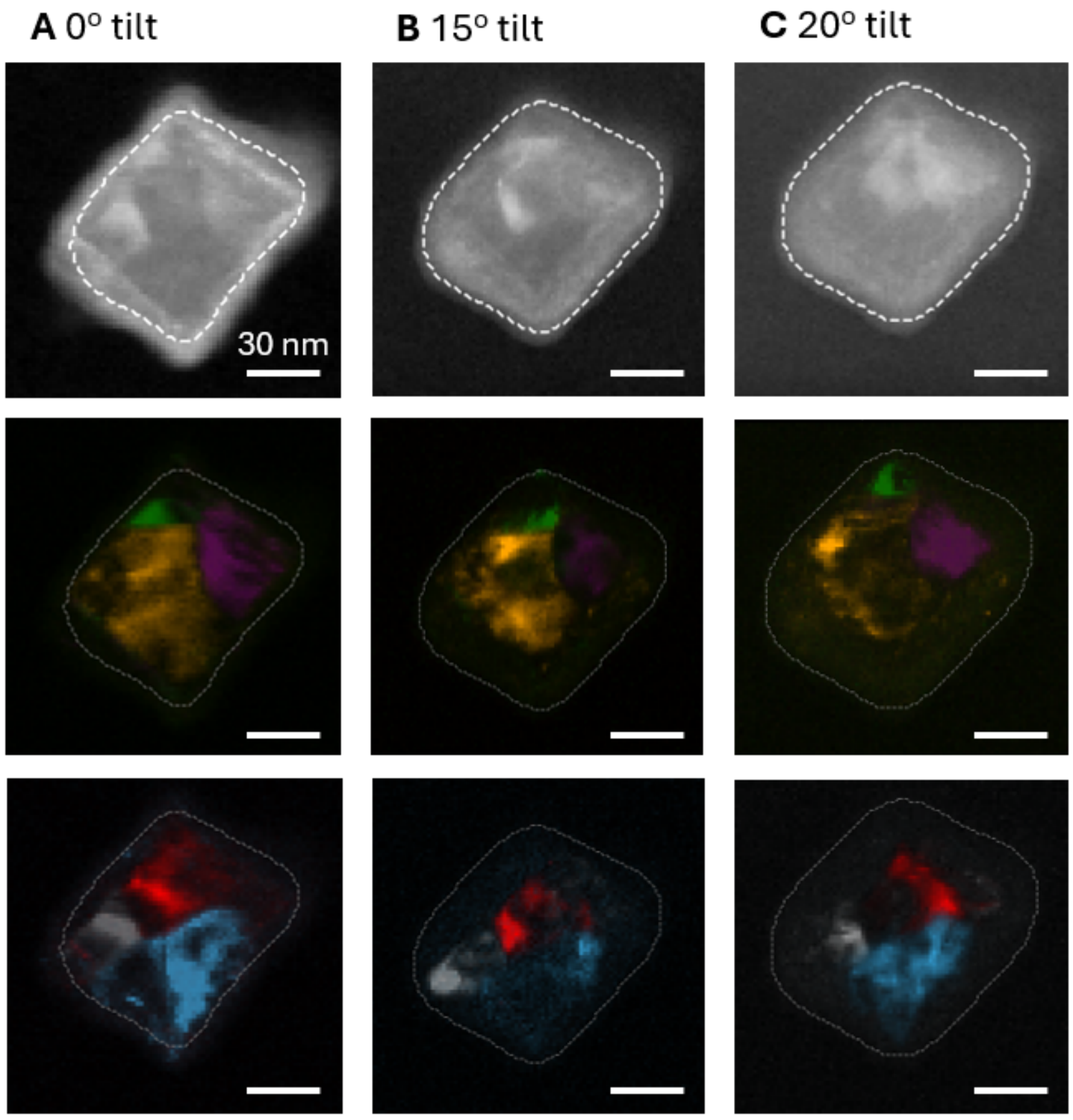


Figure S22. Virtual dark field images at various tilt angles for the ~67 nm cube in Figure 5. Total integrated intensity image (top row) and virtual dark field images (bottom rows) split in two sets of three units for clarity, color coded according to Figure S17 for A) 0°, B) 15°, and C) 20° tilt angles. The dashed white line serves as a visual outline of the cube. Virtual dark field image intensities adjusted for best visualization.

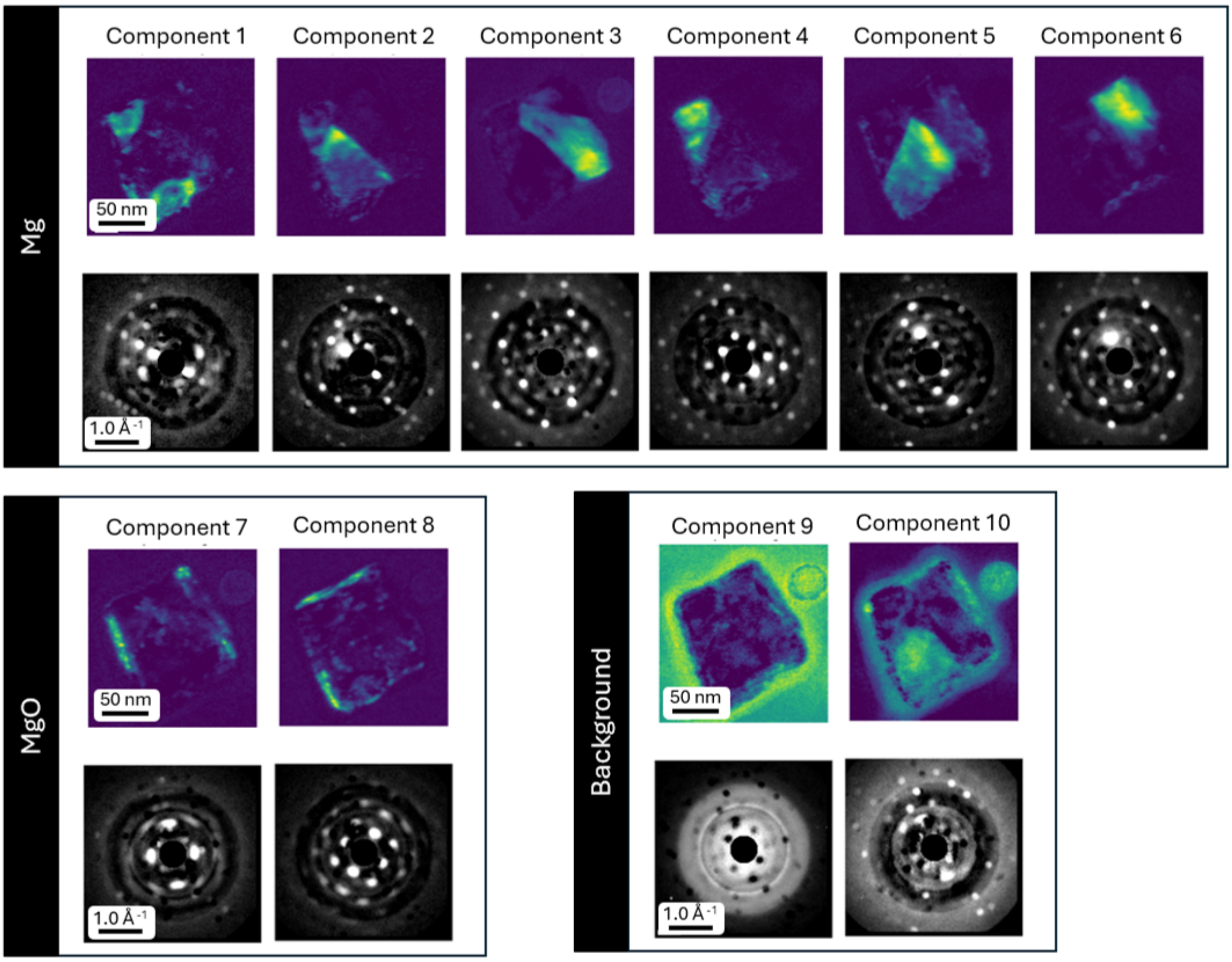


Figure S23. NMF components extracted from the ~76 nm cube 4D STEM data set in Figure 5, grouped into Mg, MgO, and background contributions. For each component, the corresponding real-space loadings (top) and reciprocal-space factors (bottom) are displayed. Components within each group are shown in no particular order.

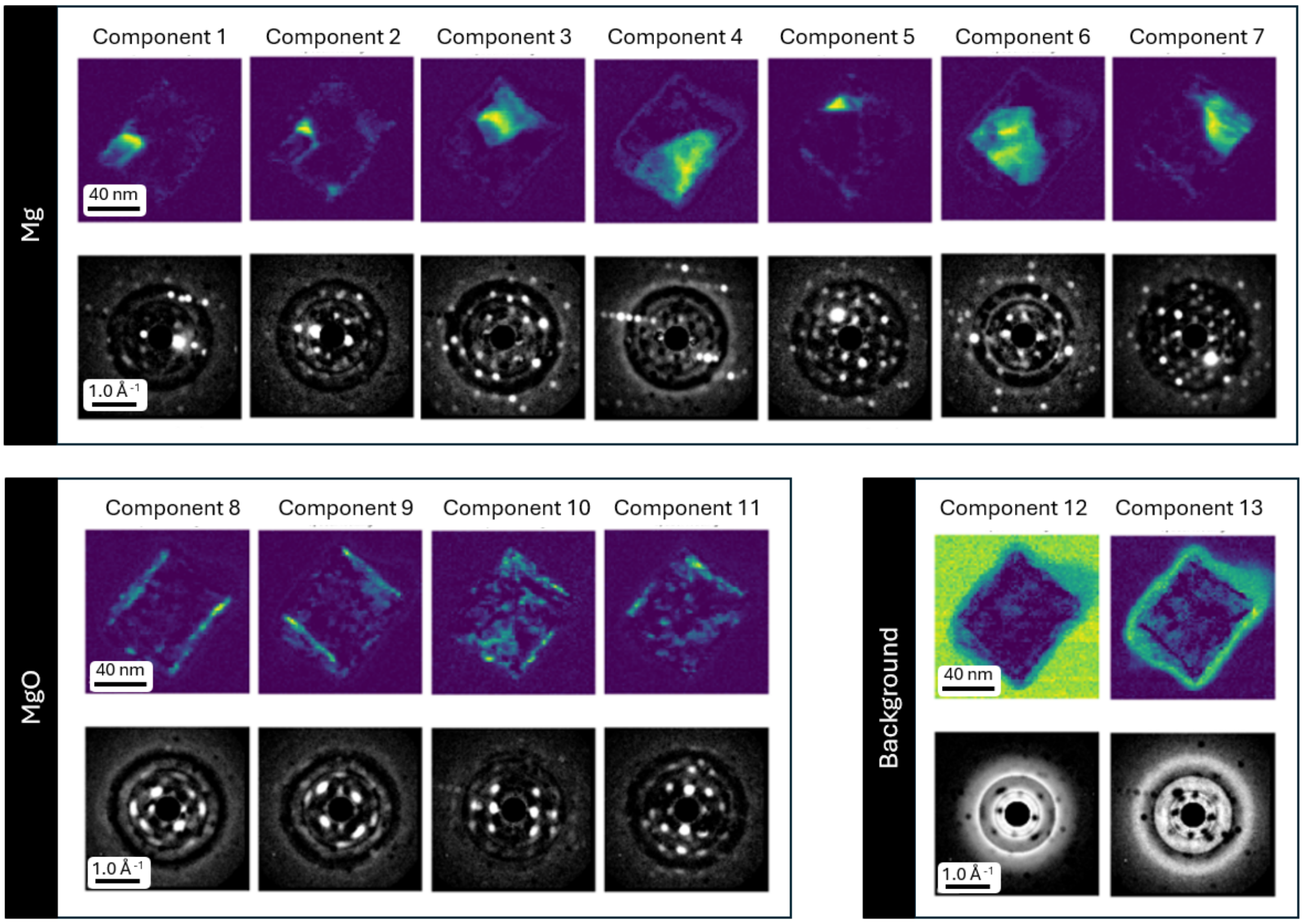


Figure S24. NMF components extracted from the ~67 nm cube in Figure 5, grouped into Mg, MgO, and background contributions. For each component, the corresponding real-space loadings (top) and reciprocal-space factors (bottom) are displayed. Components within each group are shown in no particular order.

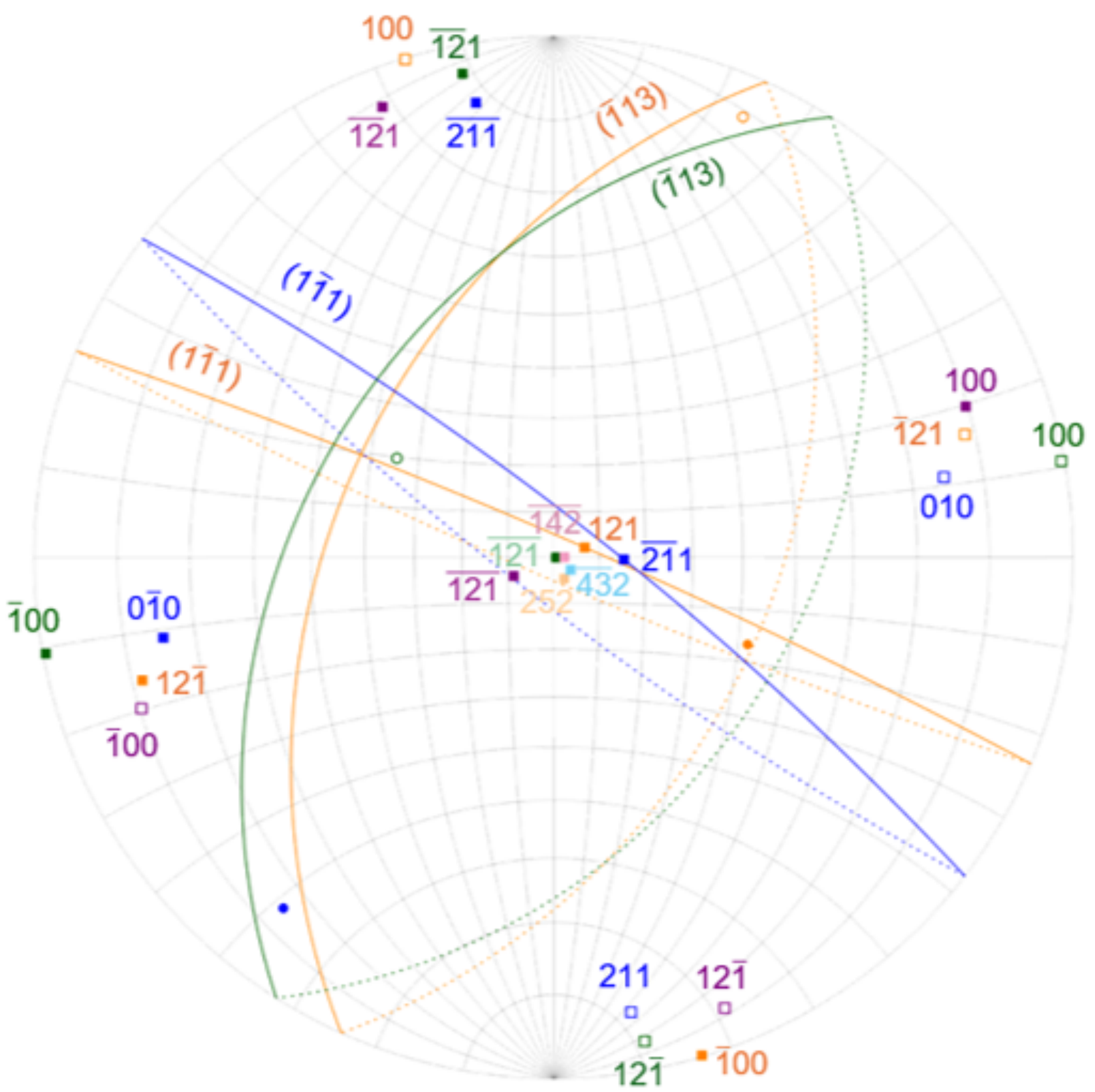


Figure S25. Stereogram showing the single crystal units in Figure S20. Squares represent crystallographic direction poles; filled squares correspond to poles in the north hemisphere and empty squares to poles in the south hemisphere. The four colors (green, red, blue, and purple) correspond to the color-coded crystallographic units in Figure S20. The pastel-colored directions in the middle of the stereogram (<$\bar{1}\bar{2}\bar{1}$>, <$\bar{1}\bar{4}\bar{2}$>, <$\bar{4}\bar{3}2$> and <252>) correspond to the experimentally assigned viewing directions, while the bold colored directions correspond to <100> and <121>-type directions that could satisfy the orthogonality of the cube facets. The plane traces, also color coded, represent twin plane candidates for twinning between two single crystalline pairs; $(1\bar{1}1)$ between the orange and the blue units and $(\bar{1}13)$ between the orange and the green units.

REFERENCES


(1) Kumar, A.; Villarreal, E.; Zhang, X.; Ringe, E. Micro-Extinction Spectroscopy (MExS): A Versatile Optical Characterization Technique. *Adv. Struct. Chem. Imaging* **2018**, *4* (1), 8. https://doi.org/10.1186/s40679-018-0057-6.

(2) de la Peña, F.; et al. Hyperspy/Exspy: V0.3.1. Zenodo 2024. https://doi.org/10.5281/zenodo.13940697.

(3) Pedregosa, F.; Varoquaux, G.; Gramfort, A.; Michel, V.; Thirion, B.; Grisel, O.; Blondel, M.; Prettenhofer, P.; Weiss, R.; Dubourg, V.; Vanderplas, J.; Passos, A.; Cournapeau, D.; Brucher, M.; Perrot, M.; Duchesnay, E. Scikit-Learn: Machine Learning in Python. *J. Mach. Learn. Res.* **2011**, *12* (85), 2825–2830.

(4) Draine, B. T.; Flatau, P. J. Discrete-Dipole Approximation For Scattering Calculations. *J. Opt. Soc. Am. A* **1994**, *11* (4), 1491. https://doi.org/10.1364/josaa.11.001491.

(5) Flatau, P. J.; Draine, B. T. Fast near Field Calculations in the Discrete Dipole Approximation for Regular Rectilinear Grids. *Opt. Express* **2012**, *20* (2), 1247–1252. https://doi.org/10.1364/OE.20.001247.

(6) Biggins, J. S.; Yazdi, S.; Ringe, E. Magnesium Nanoparticle Plasmonics. *Nano Lett.* **2018**, *18* (6), 3752–3758. https://doi.org/10.1021/acs.nanolett.8b00955.

(7) Bigelow, N. W.; Vaschillo, A.; Iberi, V.; Camden, J. P.; Masiello, D. J. Characterization of the Electron- and Photon-Driven Plasmonic Excitations of Metal Nanorods. *ACS Nano* **2012**, *6* (8), 7497–7504. https://doi.org/10.1021/nn302980u.

(8) Pehlivan, Z. S.; Ten, A.; Wayman, T. M. R.; Ringe, E. Plasmonic Magnesium Arrays with Nanosphere Lithography. *APL Photonics* **2024**, *9* (7), 071302. https://doi.org/10.1063/5.0210650.
